# Nitric Oxide‐Driven Nanomotor for Deep Tissue Penetration and Multidrug Resistance Reversal in Cancer Therapy

**DOI:** 10.1002/advs.202002525

**Published:** 2020-12-18

**Authors:** Mi Mi Wan, Huan Chen, Zhong Da Wang, Zhi Yong Liu, Yue Qi Yu, Lin Li, Zhuo Yue Miao, Xing Wen Wang, Qi Wang, Chun Mao, Jian Shen, Jia Wei

**Affiliations:** ^1^ National and Local Joint Engineering Research Center of Biomedical Functional Materials School of Chemistry and Materials Science Nanjing Normal University Nanjing 210023 China; ^2^ The Comprehensive Cancer Centre of Nanjing Drum Tower Hospital The Affiliated Hospital of Nanjing University Medical School Nanjing 210008 China

**Keywords:** cancer therapy, deep‐penetration, degradation of tumor extracellular matrix, multidrug resistance, nanomotors, nitric oxide

## Abstract

Poor permeation of therapeutic agents and multidrug resistance (MDR) in solid tumors are the two major challenges that lead to the failure of the current chemotherapy methods. Herein, a zero‐waste doxorubicin‐loaded heparin/folic acid/l‐arginine (HFLA‐DOX) nanomotor with motion ability and sustained release of nitric oxide (NO) to achieve deep drug penetration and effective reversal of MDR in cancer chemotherapy is designed. The targeted recognition, penetration of blood vessels, intercellular penetration, special intracellular distribution (escaping from lysosomes and accumulating in Golgi and nucleus), 3D multicellular tumor spheroids (3D MTSs) penetration, degradation of tumor extracellular matrix (ECM), and reversal of MDR based on the synergistic effects of the motion ability and sustained NO release performance of the NO‐driven nanomotors are investigated in detail. Correspondingly, a new chemotherapy mode called recognition‐penetration‐reversal‐elimination is proposed, whose effectiveness is verified by in vitro cellular experiments and in vivo animal tumor model, which can not only provide effective solutions to these challenges encountered in cancer chemotherapy, but also apply to other therapy methods for the special deep‐tissue penetration ability of a therapeutic agent.

## Graphical abstract

Deep drug penetration and effective reversal of multidrug resistance in cancer chemotherapy can be efficiently realized by motion ability and sustained release of nitric oxide (NO) by NO‐driven nanomotor.

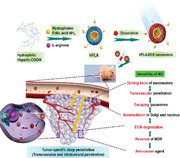

## 1 Introduction

One of the major bottlenecks of cancer chemotherapy methods which lead to treatment failure is that most of the therapeutic agents cannot reach the inner tumor tissues due to their limited penetration ability,^[^ ^1^, ^2^, ^3^, ^4^ ^]^ which is mainly caused by the complex internal structure of tumor tissue, such as high interstitial pressure, tumor solid stress, and tumor stroma.^[^ ^5^ ^]^ Among them, the existence of the ECM mainly comprised of collagen has been regarded as the key barrier that should be conquered.^[^ ^6^ ^]^ Besides, MDR of cancer cells may also appear simultaneously in the process of chemotherapy, making the treatment situation even more complicated.^[^ ^7^, ^8^, ^9^ ^]^ So far, it has been reported that more than 90% of cancer deaths are affected by MDR, which is primarily caused by the overexpression of certain proteins such as P‐glycoprotein (P‐gp) on cell membranes that excrete antineoplastic drugs.^[^ ^10^ ^]^ Therefore, solving the above problems to improve cancer cure rate becomes the research focus of the biomedical science field.

Currently, many researchers have used different strategies including internal (e.g., intratumoral pH values or overexpressed proteins) and external stimuli (e.g., ultrasound, radiation, and hyperthermia) to improve the tumor penetration of therapeutic agents.^[^ ^11^, ^12^, ^13^, ^14^ ^]^ However, the former may face the challenge that the different tumor types have different internal stimuli, which need to be studied and dealt with separately. And the weak tissue penetration of external stimuli also limits the systemic effect of the latter strategy.^[^ ^15^ ^]^ Most recently, viruses, pathogens, and lipoproteins have been utilized as natural nanocarriers owing to their high‐density lipoproteins, which are endogenous proteins that can be used to deliver different drugs because they can promote drug permeability in tumors.^[^ ^16^, ^17^, ^18^ ^]^ Yet, their clinical applications are still disputed, which may be attributed to the possibly occurred endogenous protein instability or immune disorders.^[^ ^15^ ^]^ In particular, most of the above strategies are to solve the problem of the cell membrane or intercellular permeability, and only a few studies involve strategies for the degradation of ECM outside the cancer cells. As for the reverse of MDR of cancer cells, based on the fact that the phenomenon of MDR is due to the increased expression of certain protein in cell membrane, which has the function of pumping out drugs, leading to drugs being blocked or discharged before reaching the intracellular target.^[^ ^19^ ^]^ The traditional way to solve this problem is to inhibit the MDR pumping effect or use nanocarriers to load a large number of drugs, which often brings unnecessary toxic substances into the organism.^[^ ^20^ ^]^ Besides, the problem of poor drug permeability also limits the effect of reversing MDR.

It is quite necessary to design a new therapy system that can simultaneously solve the above problems for cancer chemotherapy. The special physiological function of NO draws our attention to this field. As a signal molecule produced by organism body, NO has been selected as the molecule of the year in 1992 owing to its specific physiological and pathological function.^[^ ^21^ ^]^ In particular, previous studies have shown that NO can react with superoxide anion in the tumor environment to form oxidant peroxynitrite (ONOO^−^), so as to stimulate the production of active matrix metalloproteinases (MMPs) in the tumor stroma which can degrade almost all collagen components in ECM, improving the penetration of the therapeutic agent in the tumor site.^[^ ^6^ ^]^ In addition, NO can also be used as an effective reverser of the MDR of the tumor by inhibiting P‐gp expression.^[^ ^22^ ^]^ Yet, the application of NO has been greatly limited by its short half‐time and high sensitivity to the biological environment.^[^ ^23^ ^]^

Our previous work reported a novel NO‐driven nanomotor, which can provide a continuous supply of NO to overcome its short half‐time and has the ability to move in cellular environment.^[^ ^24^ ^]^ Especially, nanomotors with self‐propelled motion ability display rapid cell internalization owing to their cell friendly surface chemistry and fast movement inside or across cells that are driven by their fuels.^[^ ^25^, ^26^, ^27^, ^28^ ^]^ Besides, such strategies depending on physical motion behavior to promote intracellular access may overcome the heterogeneous property of different tumors and cause no immune disorders, making them among the most proper candidates to accomplish the above tasks. This prompted us to design a new kind of NO‐driven nanomotor to solve the existing problems of poor drug permeability, MDR, and unstable supply capacity of NO for cancer chemotherapy simultaneously, which has never been reported before.

**Figure 1:**
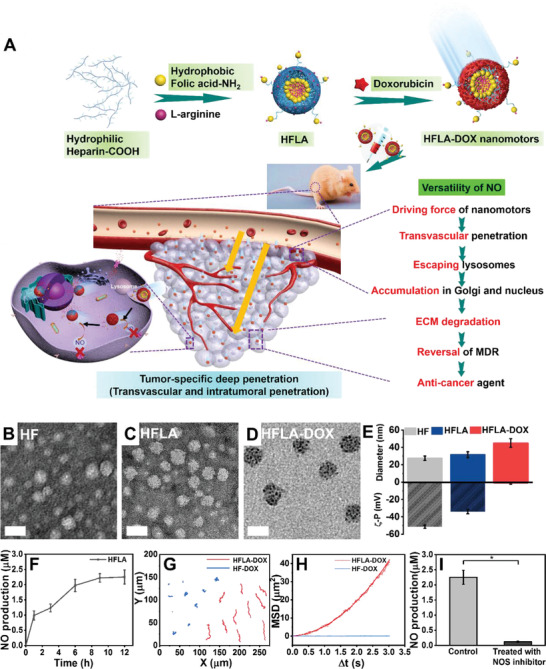
Schematic illustration, characterization, and movement behavior of the nanomotors. A) The fabrication process of HFLA‐DOX nanomotors and the versatility of NO; TEM images of B) HF, C) HFLA, and D) HFLA‐DOX (B and C were negatively stained with uranyl acetate, Scale bar: 50 nm); E) Zeta potential and nanoparticle size of HF, HFLA, and HFLA‐DOX; F) NO release profile of HFLA nanomotors in MCF‐7/ADR cellular environment; G) Representative trajectories of the HF‐DOX and HFLA‐DOX nanomotors under MCF‐7/ADR over 30 s, and H) Averaged MSDs of the HF‐DOX and HFLA‐DOX nanomotors obtained from the optical tracking. Experimental data are mean ± SD of samples in a representative experiment (*n* = 10). I) NO production by HFLA nanomotors under the MCF‐7/ADR cellular environment in the absence or presence of total NOS inhibitor treatment 12 h; Experimental data are mean ± SD of samples in a representative experiment (*n* = 3).

Specifically, we choose the heparin/folic acid (HF) nanoparticle (anticancer drug and cancer cell‐targeting agent) with a cage‐like structure as carrier to load l‐arginine and anticancer drug doxorubicin (DOX), forming HFLA‐DOX nanomotors (**Figure** **1**A), which can target the cancer cell by reacting with the overexpressed folic acid receptors.^[^ ^29^ ^]^ This is while l‐arginine can effectively promote cellular endocytosis of nanomotors by forming double‐ligand hydrogen bonds with cell membranes.^[^ ^30^ ^]^ Then, owing to the higher concentration of NO synthetase (NOS) and reactive oxygen species (ROS) in a tumor environment than normal cells,^[^ ^24^ ^]^ HFLA nanomotors can produce NO to drive the nanomotors, facilitating their intracellular distribution and intercellular transportation process. In the meantime, the produced NO can form ONOO^−^ to promote the degradation of collagen components in ECM,^[^ ^6^ ^]^ further promoting extracellular penetration of nanomotors in tumor tissues. Subsequently, the MDR of cancer cells can be reversed by NO and the released DOX can further efficiently complete the anticancer effect, thereby achieving better chemotherapeutic effect. Besides, anticancer drug HF and NO with a higher concentration can also assist DOX in killing cancer cells during this process.

The important and complex effectiveness of NO in chemotherapy have been proposed by researchers for a long time.^[^ ^31^, ^32^, ^33^, ^34^, ^35^ ^]^ Table S1, Supporting Information, summaries the current methods to investigate the possible application of NO in chemotherapy, including vascular permeability, anticancer effect, tumor ECM degradation, reversal of tumor MDR, etc. It can be found that most of the current researches about chemotherapy with NO are limited to some of these functions, and almost no literature studies more than two of the above functions at the same time. To study the specific mechanism of NO in solving the above bottleneck problems systematically, this work proposes a detailed characterization strategy from the stage of the nanocarriers entering the blood environment to the tumor ablation process. The whole process includes targeted recognition, penetration of blood vessels, intercellular penetration, intracellular distribution (escaping from lysosomes and accumulating in Golgi and the nucleus), 3D MTSs penetration, detailed mechanism of tumor tissue ECM degradation, and in vivo penetration. We fully demonstrate the role of NO and the effect of motion ability of HFLA‐DOX nanomotors during this process, which may provide a scientific strategy and method to study the effectiveness of nanomotors in cancer therapy.

## 2 Results and Discussion

HF nanoparticles were synthesized according to the literature (Figure S1, Supporting Information).^[^ ^29^ ^]^ The chemical structure of HF can be verified by ^1^H NMR spectrum (Figure S2, Supporting Information) and the mass ratio of l‐arginine in HFLA nanomotor is about 10% (Figure S3, Supporting Information). Transmission electron microscopy (TEM) results display that the average particle size of HF is about 30 nm (Figure 1B). HFLA shows similar morphology to HF with an average particle size of about 35 nm, which changes to about 50 nm after loading DOX (HFLA‐DOX) (Figure 1C,D). The possible combination mechanism of HF with l‐arginine and DOX was illustrated (Figure 1A) and verified by zeta potential, Fourier transform infrared (FTIR), and X‐ray photoelectron spectroscopy (XPS) results (Figure 1E and Figures S4–S6, Supporting Information; detailed analysis can be found in the Supporting Information). In the meantime, the successful loading of DOX in HFLA can be verified by their UV–vis spectra (Figure S7, Supporting Information) in which two main peaks located at around 280 and 500 nm appeared for HFLA‐DOX, indicating the existence of HF and DOX.

Then, we tested the duration of NO release from nanomotors under a cellular environment. The results display that the HFLA nanomotors can release NO for at least 12 h (Figure 1F). Besides, the release performance of DOX from HFLA‐DOX was also detected, which displays that the release can last for at least 48 h (Figure S8, Supporting Information). Studying the movement of nanomotors in cellular environment is rather important to predict the possibility of its future movement in vivo. The nanomotors in the MCF‐7/ADR cells display obvious motion behavior (Figure S9A and Movie S1, Supporting Information). Further, we used 3D MTSs to investigate the movement behavior of nanomotors in a 3D cellular environment, which displayed that nanomotors exhibit obvious motion behavior under 3D MTSs condition (Figure S9B and Movie S2, Supporting Information), providing a reference for the better prediction of their movement in a solid tumor environment. We also used an optical microscope to collect the video under a cellular environment, and the movement behavior and the mean squared displacement (MSD) were also analyzed. The acquired video was traced to the trajectory of the motion, and the MSD was linearly and parabola fitted based on the trajectory to determine the type of motion of different types of particles. The results are shown in Figure 1G,H. For HFLA‐DOX nanomotors, the MSD plots can be in good accordance with the parabola (*R* ^2^ = 0.999), indicating that the motion of nanomotors can be regarded as a self‐propulsion process.^[^ ^36^ ^]^

Further, in order to investigate the important role of NOS in the cellular environment for the production of NO, we used total NOS inhibitors ((S,E)‐2‐amino‐5‐(2‐methylguanidino) pentanoic acid compound with acetic acid, l‐NMMA acetate) to treat the cancer cells. It was found that the amount of NO production was obviously decreased, and the movement of the nanomotor was also notably suppressed (Figure S9C and Movie S3, Supporting Information), indicating that the existence of NOS in cells play an important role for NO production and motion ability of the nanomotors (Figure 1I).

The biocompatibility of the nanomotors was also investigated, which shows that the hemolysis rates of HF and HFLA are below 2% (Figure S10, Supporting Information), indicating their good biocompatibility. In the meantime, it has been regarded that the electronegative property of these nanoparticles can protect them from being swallowed by macrophages.^[^ ^37^ ^]^ The low uptake of the nanomotors by macrophages was also confirmed by results in Figures S11 and S12, Supporting Information, in which the nanomotors display low cytotoxicity to macrophages and few nanomotors (blue color) can be engulfed by macrophages during the incubation process (1 h).

The above results imply that the nanomotors proposed in this work may be ideal for the sustained delivery of NO and drugs into tumor.

Based on the fact that HF of the nanomotors can target the cancer cells by reacting with the overexpressed folic acid receptors in their membranes, the targeting ability of the nanomotors to cancer cells were detected. The overexpressed folate receptor in cancer cells were assessed by western blots and immunofluorescence assay. As shown in Figures S13 and S14, Supporting Information, the folate receptor protein (MW = 38 kDa) was highly expressed in MCF‐7/ADR cells, whereas it was lowly expressed in normal cells such as human umbilical vein endothelial cells (HUVECs). The nanomotors with (called HFLA) and without (called HLA) targeting groups folic acid^[^ ^24^ ^]^, cells (MCF‐7/ADR cells) with or without (HUVECs) overexpressed folic acid receptors were used in this part. The results suggest that folic acid in HFLA nanomotors may facilitate them to target cancer cells (Figures S15 and S16 and Movie S4, Supporting Information; detailed analysis can be found in Supporting Information).

**Figure 2:**
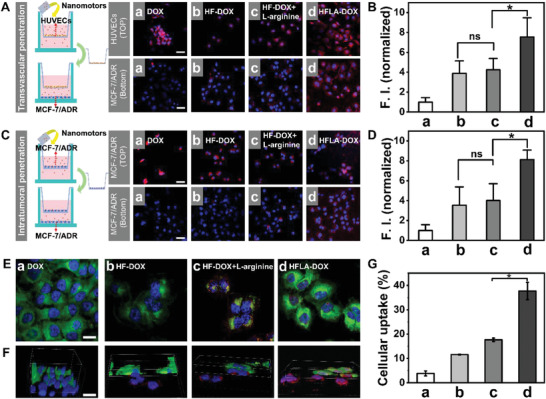
Characterization of cell penetration of nanomotors under 2D planar cellular condition. A–D) Schematic illustration and CLSM images for in vitro experimental models of transvascular extravasation and intratumoral penetration and corresponding fluorescence intensity (F.I.): a) DOX, b) HF‐DOX, c) HF‐DOX+l‐arginine, and d) HFLA‐DOX; (blue: nucleus; red: nanomotors) (Scale bar: 50 µm); E) CLSM images of MCF‐7/ADR cells after uptaking the nanomotors under different conditions: a) DOX, b) HF‐DOX, c) HF‐DOX+l‐arginine, and d) HFLA‐DOX (Scale bar: 20 µm); F) a snapshot of a 3D rendered movie (Movie S5, Supporting Information) made from a stack of confocal images (Scale bar: 20 µm), and G) corresponding cellular uptake efficiency; (blue: nucleus; green: cell membrane; red: nanomotors). Asterisk (*) denotes statistical significance between bars (*p < 0.05) using one‐way ANOVA analysis. All data are shown as mean ± SD of samples in a representative experiment (*n* = 3).

The above results demonstrate the good targeting ability of the nanomotors designed in this work. After the nanomotors reach the tumor site through intravenous administration, the next important thing is for it to enter the tumor through the blood vessels effectively. Thus, the following experiments were designed to confirm the ability of these nanomotors to penetrate from blood vessels into tumor tissues and from the outer cells of the tumor to the deeper cells of the tumor (transvascular and intratumoral penetration ability)^[^ ^38^ ^]^ (**Figure** **2**A–D; detailed analysis can be found in Supporting Information). In order to study the role of the motion effect of nanomotors during this process, the following four groups of samples were set: DOX, HF‐DOX without NO release and motion ability, HF‐DOX+l‐arginine with NO release but without motion ability, and HFLA‐DOX with both NO release and motion ability (Table S2, Supporting Information). The results depict that very weak red signals representing DOX can be detected in the lower chamber in HF‐DOX+l‐arginine group, indicating little effect of NO on the penetration of drugs between cells (Figure 2A,B and Figure S17, Supporting Information). On the contrary, enhanced red fluorescence signals can be observed for HFLA‐DOX group, implying that the motion behavior can facilitate the penetration from HUVECs to MCF‐7/ADR cells. Moreover, the promotion effect of motion ability to intratumoral penetration of the nanomotors is also confirmed by the Transwell model with both the insert and bottom chamber seeding MCF‐7/ADR cells (Figure 2C,D and Figure S18, Supporting Information; detailed analysis can be found in Supporting Information).

**Figure 3:**
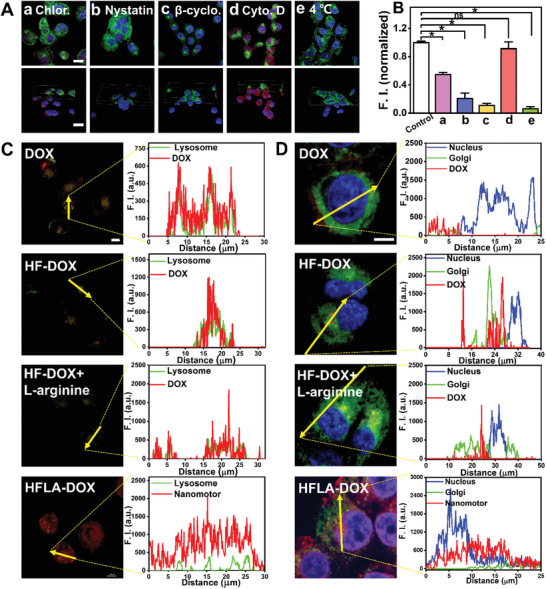
Endocytic pathway and intracellular distribution of nanomotors. A) CLSM images (Movie S6, Supporting Information) of MCF‐7/ADR cells after being incubated with nanomotors for 4 h in the presence of different endocytic inhibitors (a: chlorpromazine (chlor.), b: nystatin, c: *β*‐cyclodextrin (*β*‐cyclo.), d: cytochalasin D (cyto. D), e: 4 °C) (Scale bar: 20 µm), and B) corresponding fluorescence signal intensity; Co‐localization of nanomotors (red) with C) lysosome (green, 5 min) or D) nucleus (blue)/Golgi (green) (2 h) after being cultured with MCF‐7/ADR cells and their corresponding fluorescence intensity profiles across the cell along the selected lines (indicated by a yellow line in the image) (Scale bar: 10 µm). Asterisk (*) denotes statistical significance between bars (*p < 0.05) using one‐way ANOVA analysis. Experimental data are mean ± SD of samples in a representative experiment (*n* = 3).

As efficient uptake by cells is a prerequisite for penetration, so we need to evaluate the uptake behavior of cells to nanomotors and the main pathway for the nanomotors to enter cells. It can be seen clearly that very weak red fluorescence (DOX) appears in the cells for the DOX group (Figure 2E–G and Figure S19, Supporting Information). In contrast, the intensity of the red fluorescence signal representing nanomotors increases for cells treated with HFLA‐DOX nanomotors. Moreover, the red nanomotors can be gradually exposed by cutting the green channel of the membrane, verifying the intracellular localization of the HFLA‐DOX nanomotors (Figure 2F and Movie S5, Supporting Information). In order to describe the effect of the nanomotor's movement effect on the cellular uptake behavior more accurately, we quantitatively measured the cellular uptake efficiency of the different samples. As shown in Figure 2G, the cellular uptake efficiency of DOX was about 3.9%. After being loaded in HF, the uptake efficiency of the cell can be increased to 11.6%. The nanomotor with motion ability reflects the best promotion effect, and the efficiency of the cellular uptake was increased to 37.7%. In particular, the main pathway of nanomotors entering cancer cells was also investigated by using different endocytosis inhibitors to treat the cancer cells. Chlorpromazine (blocking clathrin‐mediated pathway), nystatin (blocking caveolae‐mediated pathway), *β*‐cyclodextrin (blocking both clathrin and caveolae‐mediated pathways), cytochalasin D (macropinocytosis inhibitor), and 4 °C (energy inhibitor) were chosen to pretreat cancer cells for 2 h before treatment with nanomotors.^[^ ^39^ ^]^ It can be seen clearly that very weak red fluorescence signals appears in cells after being treated with chlorpromazine, nystatin, and *β*‐cyclodextrin and the relative fluorescence signals decrease to about 0.5, 0.2, and 0.1, respectively (**Figure** **3**A,B; Figure S20 and Movie S6, Supporting Information). A similar intensity of red fluorescence signals can be found in cancer cells treated with cytochalasin D. The above results indicate that the main pathways for the nanomotors entering cancer cells may be dependent on both clathrin and caveolae‐mediated pathways instead of micropinocytosis.

As intracellular distribution is a key factor in the transport and effectiveness of nanomotors, thus, we studied the distribution of nanomotors in cells after they entered the cells. The co‐localization of DOX/nanomotors and lysosomes after being treated with for 5 min illustrates that almost all the internalized DOX is located in the lysosomes (Figure 3C and Figure S21, Supporting Information; detailed analysis is shown in Supporting Information). In contrast, the fluorescence curve of HFLA‐DOX in cells shows a low correlation with that of lysosomes, revealing that the nanomotors proposed in this work could readily overcome the main biological barriers of cancer cells, thus offering a much higher drug delivery ability. Further, results for co‐localization of the nanomotors with Golgi (main organelle involved in cell transport) and nucleus (main organelle where DOX kills cancer cells by destroying the tertiary structure of its DNA) reveal that the nanomotors can accumulate in both Golgi and nucleus (Figure 3D and Figure S22, Supporting Information; detailed analysis is shown in Supporting Information).^[^ ^40^, ^41^ ^]^ The distribution in Golgi may facilitate the effective transportation of nanomotors outside the cells (exocytosis), and then use the movement of the nanomotors to promote their endocytosis and exocytosis by the next adjacent cell, effectively completing cell‐to‐cell transmission.

It is worth noting that by comparing the HF‐DOX+l‐arginine group with nanomotor group (group (c) and (d) in Figure 3C,D), it can be seen that such special intracellular distribution is inseparable from the motion ability of the nanomotor. The enhancement of cellular endocytosis by nanomotors may be owing to their targeting and motion ability in cancer cells (Figure S23 and Movie S7, Supporting Information), while the inhibition of macrophage phagocytosis may be attributed to lacking targeting and motion ability of the nanomotors in macrophages (Figures S24 and S25 and Movie S8, Supporting Information; detailed analysis can be found in Supporting Information).

**Figure 4:**
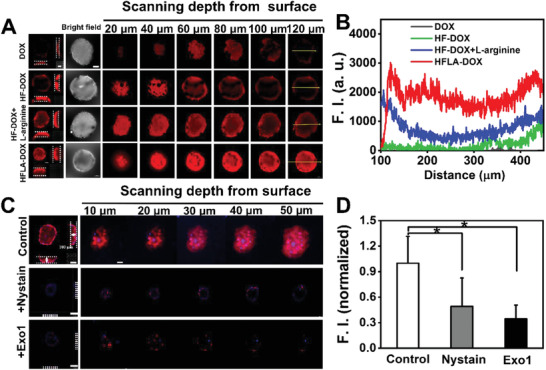
Characterization of cell penetration of nanomotors under 3D MTSs condition. A) In vitro penetration into MCF‐7/ADR MTSs after treated with different samples for 4 h, and Z‐stack pictures were taken from top to equatorial plane of MTSs in 20 µm thickness (Scale bar: 100 µm) and B) their corresponding fluorescence intensity profiles; C) CLSM images of MTSs treated with HFLA‐DOX nanomotor to investigate the influence of the caveolae‐mediated endocytosis inhibitor nystain and exocytosis inhibitor Exo1 on penetration into MCF‐7/ADR MTSs and Z‐stack pictures were taken from top to equatorial plane of MTSs in 10 µm thickness and D) corresponding fluorescence intensity. Asterisk (*) denotes statistical significance between bars (*p < 0.05) using one‐way ANOVA analysis. Experimental data are mean ± SD of samples in a representative experiment (*n* = 3).

The penetration and the intracellular distribution results of the nanomotors under a 2D cellular condition illustrates that the motion ability of nanomotors may facilitate their intercellular transportation, thus in this part, 3D MTSs were further constructed as 3D in vitro tumor models to forward the determination of the penetration ability of the nanomotors we proposed. The penetration depth (about 80 µm) and fluorescence intensity across the 120th µm section of the 3D MTSs (about 1000–2000 a.u.) for HF‐DOX+l‐arginine group are much lower than that of the nanomotor group (about 120 µm and 3000 a.u.), indicating that the motion behavior of nanomotors can greatly promote the penetration process of drugs in 3D MTSs (**Figure** **4**A,B). In the meantime, the endocytosis inhibitor Nystain and exocytosis inhibitor Exo1 were used to treat 3D MTSs to investigate a possible penetration mechanism under a 3D condition.^[^ ^42^, ^43^ ^]^ The results display that both the endocytosis and exocytosis process are suppressed significantly by the treatment of above inhibitors, further confirming the fact that the nanomotors are dependent on endocytosis into cells, and transfer them between cells through Golgi (exocytosis) in which their motion ability can effectively facilitate these processes (Figure 4C,D; detailed analysis is shown in Supporting Information).

**Figure 5:**
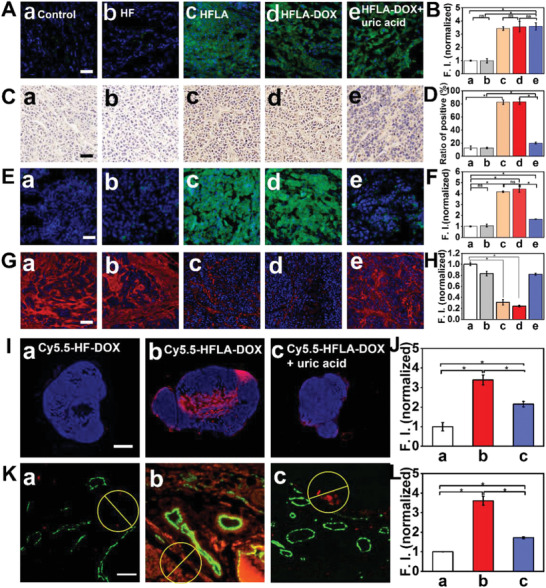
In vivo characterization of NO‐driven nanomotors generating ONOO^−^, degrading collagen, and deep‐tissue penetration. A) Fluorescence images of NO in tumor tissues using DAF‐FM DA fluorescent probe (Scale bar: 50 µm) and B) corresponding fluorescence signal intensity; C) Immunohistochemical detection of 3‐nitrotyrosine (3‐NT, brown) in tumors (purple color:nuclei stained with hematoxylin) (Scale bar: 50 µm) and D) corresponding ratio of all positive signal; E) In situ zymography assay of tumor to characterize the collagenase activity which reflects the activity of MMP (Green color is caused by the hydrolysis of DQ collagen by collagen enzymes, blue color represents cell nuclei; Scale bar: 50 µm) and F) corresponding fluorescence signal intensity; G) Immunofluorescent staining of Collagen I in tumors (Red color:collagen I; blue color:DAPI) (Scale bar: 50 µm) and H) corresponding fluorescence signal intensity; (samples for A–H: a: control, b: HF‐DOX, c: HFLA, d: HFLA‐DOX, e: HFLA‐DOX+uric acid); I) Fluorescence images of the tumor frozen section representing the penetration of cy5.5 labeled samples (red color) (Scale bar: 2 mm) and J) corresponding fluorescence signal intensity; K) Immunofluorescence images displaying the distribution of cy5.5 stained samples (red color) in tumor (blood vessels were stained with CD31 antibody, green color) (Scale bar: 50 µm) and L) corresponding fluorescence signal intensity; (samples for I–L: a: cy5.5 labeled HF‐DOX, b: cy5.5 labeled HFLA‐DOX, c: cy5.5 labeled HFLA‐DOX+uric acid). Asterisk (*) denotes statistical significance between bars (*p < 0.05) using one‐way ANOVA analysis. Experimental data are mean ± SD of samples in a representative experiment (*n* = 3).

The promotion effect of motion behavior of nanomotors on the intracellular distribution and intercellular penetration of nanomotors is carefully verified by the above results. Next, the unique role of NO in tumor penetration will be carefully characterized. As mentioned before, ONOO^−^ can be formed by the reaction of NO and superoxide anion in the tumor environment, which can stimulate the production of MMP in tumor stroma to degrade almost all collagen components (such as collagen I) in ECM.^[^ ^6^ ^]^ In order to fully study the role of NO and ONOO^−^ during this process, HFLA+uric acid group with motion ability while ONOO^−^ would be eliminated by uric acid was set for comparison (Table S2, Figure S26, and Movie S9, Supporting Information).^[^ ^6^ ^]^ The NO release in tumor tissues was assessed first. It was found that neither the control group nor the HF‐DOX group can generate obvious NO in tumor tissues due to their lack of l‐arginine. The active ingredient capable of producing NO include HFLA, HFLA‐DOX, and HFLA‐DOX+uric acid group can produce a plentiful amount of NO with similar fluorescence intensities (the fluorescence intensity is about three times that of the control group) (**Figure** **5**A,B and Figure S27, Supporting Information). Since uric acid is only a scavenger of ONOO^−^, it does not affect NO production by HFLA‐DOX nanomotors in tissues. Then we use immunohistochemical staining against 3‐nitrotyrosine (3‐NT) in proteins (biomarker of ONOO^−^ activity) to characterize the formation of ONOO^−^. An obvious brown color (representing 3‐NT) can be found in HFLA and HFLA‐DOX group instead of HFLA‐DOX+uric acid group, indicating that the ONOO^−^ scavenger uric acid can significantly suppress the ONOO^−^ formation (Figure 5C,D). Next, we detect whether the activity of MMP enzymes can be upregulated by the increased ONOO^−^ production, and one well‐investigated representative MMPs (MMP‐1 and MMP‐2) were chosen for analysis by using in situ zymography method.^[^ ^44^, ^45^ ^]^ A significantly enhanced expression and activity of the MMP can be found in HFLA and HFLA‐DOX groups while a low activity of MMP is found in HFLA‐DOX+uric acid group (Figure 5E,F and Figures S28 and S29, Supporting Information). Finally, the degradation of ECM was detected. It has been recognized that collagen I is the main component in ECM to inhibit the deep penetration of nanotherapeutic agents for cancer therapy.^[^ ^46^ ^]^ Here, the in vivo degradation of collagen I was characterized. The results display that a very weak fluorescence signal representing collagen I can be found in tumor treated with HFLA or HFLA‐DOX with ONOO^−^ formation, and only 20% of the original fluorescence intensity is retained, indicating the depletion of collagen I (Figure 5G,H and Figure S30, Supporting Information). On the contrary, uric acid treated HFLA‐DOX can no longer degrade collagen I in vivo owing to the inhibition effect of uric acid for the formation of ONOO^−^.

The above results confirm the unique effectiveness of motion ability and NO production of the nanomotors on promotion of cellular uptake, intracellular distribution, intercellular transport, and tumor ECM degradation ability using cellular and in vivo model, respectively. In this part, the synergistic promotion effect of motion ability and NO of the nanomotors proposed in this work to the deep penetration in tumor tissue will be demonstrated by in vivo characterizations. As shown in Figure 5I,J, there is a small amount of red fluorescence signal (relative fluorescence intensity is denoted as 1) in the tumor slices treated by the pure HF‐DOX, most of which is concentrated on the edge of the tumor tissue, indicating limited penetration ability of the pure HF‐DOX without motion ability and ONOO^−^ production ability. The HFLA‐DOX group shows superior penetration ability, almost all the inside of the tumor is filled with red fluorescent signal, and the signal intensity is about three times that of the HF‐DOX group. The group of HFLA‐DOX+uric acid shows a certain degree of reduced penetration intensity (relative fluorescence signal intensity is about 2), but it is still stronger than the fluorescence intensity of the HF‐DOX group, implying that the motion behavior of the nanomotors can also promote the penetration process.

Furthermore, immunofluorescence staining for the blood vessels inside the tumor to determine the penetration of the nanomotor in the tumor blood vessels was performed, and the fluorescence signal intensity of the circular area with the same distance from the blood vessels was quantified (Figure 5K,L and Figure S31, Supporting Information). The result displays that the red fluorescence signals in HF‐DOX group mostly locate around blood vessels and relative signal intensity is very weak due to lack of motion ability and ONOO^−^ production of HF‐DOX. The HFLA‐DOX group with both motion ability and ONOO^−^ exhibits much better drug diffusion performance in both amount and depth (relative fluorescence intensity is larger than 3.5). The red fluorescence signals not only appear around the blood vessel, but also penetrate to a considerable distance from the blood vessel. The uric acid‐treated group displays better penetration ability than HF‐DOX group (relative fluorescence intensity is about 1.7 times that of the HF‐DOX group) but poorer than that of HFLA‐DOX group. These results confirm the synergistic effect of the nanomotor's motion ability and the biomedical functions of NO on the deep penetration of drugs in tumor tissues.

**Figure 6:**
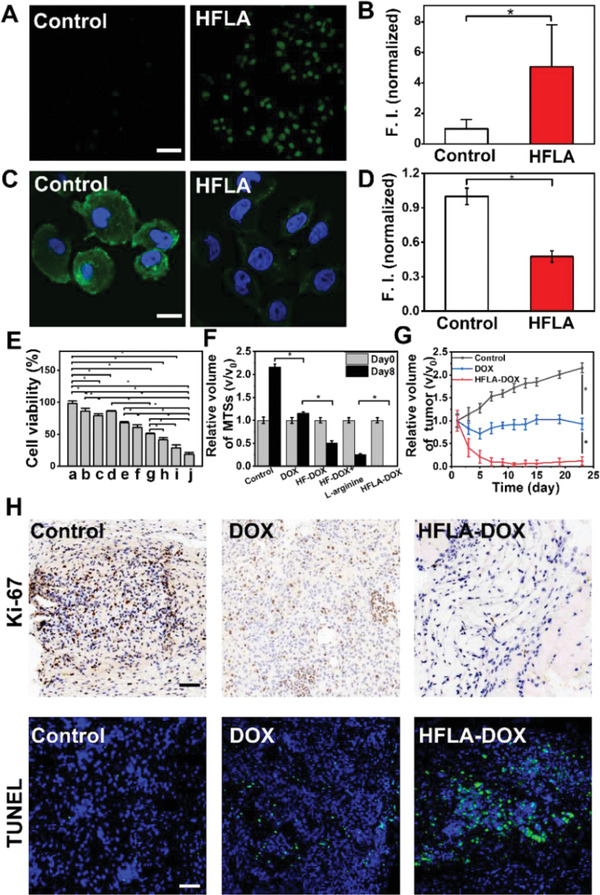
Therapy ability of the tumor cells under different conditions by the nanomotors. A,B) CLSM images recording the intracellular NO production using the NO fluorescent probe DAF‐FM DA and its corresponding fluorescent intensity for control, HFLA (Scale bar: 100 µm). C,D) P‐gp expression of MCF‐7/ADR cells using immunofluorescence staining and its corresponding fluorescent intensity for control and HFLA (Scale bar: 20 µm). E) MTT results of a) l‐arginine (100 µg mL^−1^), b) HF (100 µg mL^−1^), c) HFLA (100 µg mL^−1^), d) DOX (50 µg mL^−1^), e) HF‐DOX (50 µg mL^−1^), f) HF‐DOX+l‐arginine (100 µg mL^−1^), g) HFLA‐DOX (50 µg mL^−1^), h) HFLA‐DOX (100 µg mL^−1^), i) HFLA‐DOX (150 µg mL^−1^), and j) HFLA‐DOX (200 µg mL^−1^). F) The elimination of 3D MTSs treated under different conditions for different days; Experimental data are mean ± SD of samples in a representative experiment (*n* = 10). G) In vivo tumor growth profiles in mice treated with DOX and HFLA‐DOX nanomotors. H) Immunohistochemical results by using Ki‐67 (brown color:proliferating cells; blue color:nucleus) and immunofluorescence TUNEL tests (green color:apoptotic cells; blue color:nucleus) of tumor tissues after being treated with different samples (Scale bar: 50 µm). Asterisk (*) denotes statistical significance between bars (*p < 0.05) using one‐way ANOVA analysis. Experimental data are mean ± SD of samples in a representative experiment (*n* = 3).

The reversal of MDR of cancer cells by the produced NO was characterized in detail, including in vitro production of NO (**Figure** **6**A,B and Figure S32, Supporting Information), western blot assay (Figure S33, Supporting Information), and immunofluorescence staining (Figure 6C,D and Figure S34, Supporting Information), displaying decreased expression of P‐gp for cells treated with HFLA‐DOX nanomotors (detailed analysis is shown in Supporting Information).

In vitro antitumor results of the nanomotors illustrate that HFLA‐DOX shows the best anticancer performance to MCF‐7/ADR cells, and only 50% of the cells maintain their viability at the concentration of 50 µg mL^−1^ (24 h), which further decrease with the increasing concentration of HFLA‐DOX nanomotors (Figure 6E; Figure S35 and Table S3, Supporting Information). In the meantime, 3D MTSs were also used as a tumor model in vitro. For HFLA‐DOX nanomotors, the 3D MTSs dissociated and finally lost 3D structure, which can be eliminated within about 8 days, implying that the enhanced drug penetration into tumor spheroids by the help of the motion ability of nanomotors can promote their antitumor efficiency (Figure 6F and Figure S36, Supporting Information; detailed analysis is shown in Supporting Information). The growth of MTSs was also monitored by using live‐cell phase contrast microscope, and the density and morphology were measured using Image J software. It can be seen from Figure S37, Supporting Information, that the cellular density within MTSs treated by HFLA‐DOX nanomotors decreased a lot compared with that of MTSs treated by samples without motion ability. Moreover, we also assayed the cell activity of the above‐mentioned 3D MTSs by using MTT method. It can be seen from Figure S38, Supporting Information, that the cell activities of the MTSs for Control, DOX, HF‐DOX, HF‐DOX+l‐arginine, and HFLA‐DOX group were about 100%, 55.4%, 24.2%, and 3.0%, respectively, which were in line with the trends of Figure 6F.

The final purpose of efficient cell‐and‐tissue penetration is to overcome the challenges in systemic chemotherapy for drug‐resistance tumor. Thus, mice with MCF‐7/ADR tumors were utilized as animal models to determine in vivo antitumor ability of the nanomotors. The biodistribution of nanomotors in the main organs of mice was detected. The cy5.5‐labeled HF‐DOX and cy5.5‐labeled HFLA‐DOX were injected into the mice via the tail vein and their distribution in the major organs was observed after 24 h. As shown in Figure S39, Supporting Information, some of the fluorescence signal appeared in the liver and kidney, and the distribution of both HF‐DOX and HFLA‐DOX was lower in the heart, spleen, and lung. It can be seen from Figure 6G that the relative tumor volume decreased quite a lot for the experimental group, which decreases to about 0.05 after treatment, indicating good tumor elimination ability of HFLA‐DOX nanomotors. Yet, the weak elimination ability for drug‐resistance tumor was observed on DOX group. Moreover, the immunohistochemical investigation demonstrates much fewer Ki‐67‐positive proliferative cells (Figure 6H) while much more TUNEL‐positive apoptotic cells can be found in the tumor tissue for HFLA‐DOX group than in the DOX group, which displays apparent proliferative cells and almost no dead cells (Figure S40, Supporting Information). All the experimental mice can maintain their weight and normal tissue structure in their main organs during the therapy process (Figures S41 and S42, Supporting Information).

Based on the above results, we tentatively propose a recognition‐penetration‐reversal‐elimination model for drug‐resistant tumors based on drug‐loaded NO‐driven nanomotor (detailed discussion is shown in Supporting Information). The nanomotor first uses folic acid to target tumor sites by recognizing folic acid receptors that were overexpressed in tumor cells. Then, the specific high concentration of NOS and ROS at the tumor site could effectively trigger the movement of the HFLA‐DOX nanomotors, thereby enhancing their penetration ability in tumor cells. The released NO can reverse the MDR of the tumors, and DOX loaded on the tumors finally completes the task of eliminating the tumors. In particular, anticancer drug HF and NO with a higher concentration can assist DOX in completing the combined killing of tumors in this process.^[^ ^47^, ^48^ ^]^ It is worth emphasizing that all the above‐mentioned processes cannot be completed without the cooperation between the components of NO‐driven nanomotor (driving gas NO, fuel l‐arginine, carrier HF). That is to say, the specific components of nanomotor participate in tumor recognition (HF), facilitated penetration (l‐arginine and NO), reversal of MDR (NO), and elimination of tumor (DOX, high concentration NO and HF) process, respectively. This is also the biggest difference between the zero‐waste/biomimetic NO‐driven nanomotor proposed in this work and various types of existing nanomotors. Using the important physiological process of the human body to solve the difficulties in the treatment of diseases in vivo is the original intention of our design of a bionic zero‐waste nanomotor, and the effect of the reaction of human body itself and its raw materials can always bring us more surprises.

## 3 Conclusion

In summary, a strategy for cell‐and‐tissue deep penetration for MDR solid tumor relying on a bio‐inspired zero‐waste nanomotor (HFLA‐DOX) was constructed, and a chemotherapeutic model called recognition‐penetration‐reversal‐elimination for cancer therapy was proposed and verified in this work. These zero‐waste nanomotors had good motion ability in both aqueous condition and 2D/3D cellular environment, which can accumulate in the tumor site and be triggered by specific higher ROS and NOS concentration in tumor to produce NO as driving force for nanomotors and the reversal agent for MDR tumor. Thanks to the motion behavior of nanomotors, the versatility of NO can be fully utilized, including driving force of the nanomotors, promoting transvascular penetration, escaping lysosomes, accumulating in Golgi and nucleus, ECM degradation, reversal of MDR, and anticancer effect. In vitro and in vivo results imply that the HFLA‐DOX nanomotors proposed in this work could overcome the drug‐resistant tumor and penetration obstacles existing in current tumor treatment modalities. This work provides the illustration of zero‐waste NO‐driven nanomotors to realize the simultaneously deep cell‐and‐tissue dual‐penetration and reversal of MDR of tumor, which is different from any existing nanomotors that use physical effects to improve the penetration ability of therapeutic agents in tumors, offering possible clues for the development of a new avenue for cancer therapy.

## 4 Experimental Section

Methods and any associated references are available in the Supporting Information.

All animals received humane care in compliance with the Guide for the Care and Use of Laboratory Animals published by the National Academy of Sciences, and all experiments procedures and protocols were approved by the Animal Research Committee of Drum Tower Hospital (Nanjing, China, Ethical approval number:2020AE01002).

## Conflict of Interest

The authors declare no conflict of interest.

## Supporting information

Supporting Information

Supplemental Movie 1

Supplemental Movie 2

Supplemental Movie 3

Supplemental Movie 4

Supplemental Movie 5

Supplemental Movie 6

Supplemental Movie 7

Supplemental Movie 8

Supplemental Movie 9
