# Supplementary material for: Nitric Oxide‐Driven Nanomotor for Deep Tissue Penetration and Multidrug Resistance Reversal in Cancer Therapy
Source: Adv Sci (Weinh). 2020 Dec 18;8(3):2002525. doi: 10.1002/advs.202002525 (PMC7856908; doi:10.1002/advs.202002525)
Supplement: Supplementary file 1 — Supporting Information [file ADVS-8-2002525-s001.pdf]

WILEY-VCH

© 2020 Wiley-VCH GmbH

ADVANCED SCIENCE
Open Access

Supporting Information

for *Adv. Sci.,* DOI: 10.1002/advs.202002525

Nitric Oxide-driven Nanomotor for Deep Tissue Penetration and Multidrug Resistance Reversal in Cancer Therapy

Mi Mi Wan, Huan Chen, Zhong Da Wang, Zhi Yong Liu, Yue Qi Yu, Lin Li, Zhuo Yue Miao, Xing Wen Wang, Qi Wang, Chun Mao, Jian Shen,* and Jia Wei**

© 2020 Wiley-VCH GmbH

Supporting Information

Nitric oxide-driven nanomotor for deep tissue penetration and multidrug resistance reversal in cancer therapy

*Mi Mi Wan†1, Huan Chen†1, Zhong Da Wang2, Zhi Yong Liu1, Yue Qi Yu1, Lin Li2, Zhuo Yue Miao1, Xing Wen Wang1, Qi Wang1, Chun Mao*1, Jian Shen*1, Jia Wei*2*

Supplementary Information Text

Experimental section
Materials and Methods.

Fabrication of succinylated heparin. Succinylated heparin was prepared according to the literature.[1] Briefly, the heparin sodium (185 USP unit mg^{-1}, Aladdin Chemistry Co., Ltd., 1.00 g) dissolved in 50 mL distilled water was added to Amberlite 732 column (H^+ form, Aladdin Chemistry Co., Ltd., 200 mL) drip by drop at 4 °C. The pH value of the collected solution was adjusted to 6.0 by trinbutylamine and then freeze-dried to obtain white solid trinbutylammonium salt. Next, trinbutylammonium salt (0.50 g) was dissolved in 10 mL N, N-dimethylformamide (DMF) and cooled to 0 °C in N_2 atmosphere. Then succinic anhydride (1.30 g, Aladdin Chemistry Co., Ltd.), triethylamine (1.31 g) and 4-dimethylaminopyridine (DMAP, Aladdin Chemistry Co., Ltd., 0.04 g) were added in the reaction system, and the reaction was continued for 24 h. The product was dialyzed (Mw. 3500 Da) for 3 days and neutralized with 1.00 M NaOH. After lyophilization, succinylated heparin was obtained as a white powder.

Fabrication of heparin-folate (HF) nanoparticles. Succinylated heparin (0.40 g) was dissolved in dimethyl sulfoxide (DMSO, 10 mL) and activated at room temperature for 1.5 h after adding 1-ethyl-3-(3-dimethylaminopropyl) carbodiimide hydrochloride (EDC, Aladdin Chemistry Co., Ltd., 0.07 g) and N-hydroxysuccinimide (NHS, Aladdin Chemistry Co., Ltd., 0.02 g). After that, NH_2 modified folic acid (0.08 g, prepared according to the reference) was added to the reaction system, [2] and the reaction lasted for 24 h at 35 °C. The solution was dialyzed against deionized water for 3 days to remove excess reactants. The resulting solution passed through a cation exchange column (Amberlite 732, H^+ form, 200 mL). Then the NaOH aqueous solution (0.10 M) was added to adjust the pH value to 7.0 followed by continuous dialysis in water for 24 h. Finally, HF (yellow powder) was obtained by freeze-drying.

Fabrication of cy5.5 labeled HF. Polyoxyethylene bis(amine) (20 mg, Apexbio) was dissolved in DMSO (2 mL), and cy5.5-NHS ester (1 mg mL^{-1}, Apexbio) dissolved in 1 mL DMSO was added dropwise to prepare Cy5.5-PEG-amine at room temperature for 12 h.[3] The succinylated heparin (0.4g) was dissolved in 20 mL anhydrous DMSO, then EDC (0.065 g) and NHS (0.024 g) were added successively, and activated at room temperature for 3 h. Folate-NHS was added to the above solution under the condition of dark. After 12 h, Cy5.5-PEG-amine was added to continue the dark reaction for 12 h, cy5.5 labeled HF was obtained by freeze-drying after dialysis.

Fabrication of heparin/folic acid/L-arginine (HFLA) and drug loaded nanomotor. The reaction system contains HF aqueous solution (0.5 mg mL^{-1}) and L-arginine (Yuanye Biotechnology Co., Ltd., Shanghai, China) aqueous solution (5 mg mL^{-1}) with a volume ratio

of 1:1. HF was added to L-arginine solution dropwise under ultrasound condition. After 15 min, HFLA nanoparticles were obtained by centrifugation (13,900×g, 10 min), washing by PBS and freeze-drying. Then 400 μg of the HFLA sample was mixed with Doxorubicin (DOX, Energy Chemical Co., Ltd. China) solution (1 mg mL^{-1}, 5 mL) in a silent mixer for 24 h. After that, the HFLA-DOX nanomotor was obtained by washing for three times with PBS to remove the DOX which was not loaded. Lastly, the mixtures were centrifuged at 13,900×g for 10 min. The product was washed for three times by deionized water. HFLA-DOX labeled with cy5.5 can be prepared by replacing HF with cy5.5-HF according to the above steps.

Characterizations.

Characterization of materials. The structure of HF was characterized by ^{1}H NMR (400 MHz) spectrum recorded on a Bruker Avance 400 spectrometer (in D_2O). Morphologies of the samples were observed on JEM-2100 transmission electron microscopy (HITACHI, Japan) by using 4% uranium acetyldioxide for negative staining. Zeta potential data of samples were measured by Zetasizer Nano-Z instrument (Malvern Instruments, UK) at 25 ºC. Fourier transform infrared (FTIR) spectra were recorded on the Cary 5000 FTIR spectrophotometer (VARIAN, USA). X-ray photoelectron spectroscopy (XPS) spectra was tested on Thermo Scientific ESCALAB 250Xi. Ultraviolet (UV) absorption spectra were recorded on a PerkinElmer 650 spectrophotometer (PerkinElmer Ltd., US). Absorbance measurements for cell viability were performed using microplate reader (Multiskan FC, Thermo Fisher instruments Co., Ltd., US). All fluorescence spectra of samples measurements were accomplished on a F-4600 spectrophotometer (HITACHI, Japan). All cell imaging experiments and motion performance of nanomotors in cellular environment were carried out using confocal laser scanning microscopy (CLSM, HP Apo TIRF 100X N.A. 1.49, Nikon, Ti-E-A1R, Japan) and optical microscope (Nanjing Jiangnan Yongxin Optics Co., Ltd., XD-202).

Detection of L-arginine consumption and NO production rate. In aqueous condition, 20% H_2O_2 was used to simulate reactive oxygen species (ROS). The HFLA (100 μg mL^{-1}) and HFLA-DOX nanomotors (100 μg mL^{-1}) were reacted with ROS, respectively. The L-arginine content in the solution during the reaction process (0, 0.5, 1, 2, 3, 6, 9 h) was detected by color-developing method.[4] 5 μL of the mixture was mixed with the chromogenic solution of L-arginine containing NaOH (1 mL, 1.0 M), diacetyl/propanol (1 mL, 0.5 mL L^{-1}) and 1-naphthol/propanol (1 mL, 0.6 M) for 15 min at 30 °C. Then its absorbance was measured by UV spectrophotometer at 540 nm. The NO concentration was detected by NO kit (Nitrate reductase method, Nanjing Jiancheng Bioengineering Institute.).

***In vitro* drug release.** The HFLA-DOX nanomotor (800 μg) was dissolved in 5 mL of PBS solution. At a set time point, 4.8 mL of sustained-release solution was taken for analysis and the same volume of buffer solution was added immediately. The concentration of DOX was tested by an UV spectrophotometer at an excitation wavelength at 490 nm.

Cell culture. Michigan cancer foundation-7 cells (MCF-7, cell NO. 115), multidrug resistant (MDR) MCF-7 (MCF-7/ADR, cell NO. 115) cells and the mouse macrophages (RAW 264.7, cell NO. 264.7) were obtained from Shanghai Meixuan Biotechnology Co., Ltd.. MCF-7/ADR cells were cultured in complete Roswell park memorial institute-1640 (RPMI-1640) medium containing 10% fetal bovine serum (FBS), 1% penicillin/streptomycin and DOX (1.0 μg mL^{-1}) in a humidified atmosphere with 5% CO_2, at 37 ºC. Human umbilical vein endothelial cells (HUVECs, cell NO. CRL-1730) were purchased from American type culture collection (ATCC, U.S.). Cell culture media modified RPMI-1640, Dulbecco's modified eagle's medium (DMEM), penicillin/streptomycin, and FBS were purchased from Corning. Cells were tested for mycoplasma and no mycoplasma infection was found.

The biocompatibility of the nanomotors.

The hemolysis test. Fresh rabbit red blood cells (RBCs) were collected by removing serum from the blood under centrifugation (810×g, 10 min). L-arginine, HF and HFLA-nanomotor (100 μg mL^{-1}) were co-incubated with RBCs (2%) for 3 h at 37 ºC. Negative (PBS) and

positive (deionized water) controls were set up at the same time. The percent hemolysis is calculated by the following formula: Hemolysis (%)=(OD_t-OD_{nc})×100%/(OD_{pc}-OD_{nc}), in which OD_t, OD_{nc}, and OD_{pc} referred to the absorbance values of the testing samples, negative and positive control.

The cell viability and uptake of the nanomotors by macrophages. RAW264.7 cells (5×10^4 cell mL^{-1}) were inoculated and cultured for 12 h to adherence. After discarding the original culture medium, cells were treated with HFLA nanomotor (200 μL) with different concentrations (from 25 to 100 μg mL^{-1}) for 1 h incubation. Then cells were incubated with 3-(4, 5-dimethyl-2-thiazolyl)-2, 5-diphenyl-2-H-tetrazolium bromide (MTT, ApexBio Technology LLC, USA, 5 mg mL^{-1}) for 4 h in the dark and the produced formazan precipitates were dissolved in DMSO. The absorbance was measured at a wavelength of 570 nm by microplate reader. The experiment was repeated for three times. The RAW264.7 cells where cultured in dishes, cell membrane was stained with green fluorescence by 3, 3′-dioctadecyloxacarbocyanine perchlorate (DiO'; DiOC18, 10 μM in DMSO, Shanghai Yuanye Biotechnology Co., Ltd. China). The phagocytosis of the RAW264.7 to nanomotors was observed under the excitation wavelength of 405 nm and 488 nm by CLSM.

The motion behavior of nanomotors under cellular condition.

The motion behavior of nanomotors under 2D cellular condition. MCF-7/ADR (5×10^4 cell mL^{-1}) were inoculated in confocal dishes and cultured in incubator for 24 h. After discarding the original culture medium, 200 μL of HFLA-DOX nanomotors (200 μg mL^{-1}) were added. The motion of nanomotors was captured by AVI mode of CLSM.

The motion behavior of nanomotors under 3D multicellular tumor spheroids (MTSs). MTSs were prepared by using Corning's round bottom 96-wells plate with ultra-low attachment property. MCF-7/ADR cells (5×10^3 cells per well) were seeded into 96-wells plate to form MTSs. In this experiment, the HFLA-DOX (200 μg mL^{-1}) were added to the dishes of the MTSs. The motion of the nanomotors was captured by AVI mode of CLSM.

Targeting ability of the HFLA nanomotors.

Targeting ability of the HFLA nanomotors to cancer cells. Hyperbranched polyamide/L-arginine (HLA) nanomotor without targeting ability was prepared according to our previous reported method.[5] MCF-7/ADR cells were inoculated into 96-wells plate and confocal dishes, which were then incubated for 4 h with different samples (HFLA and HLA nanomotors, 100 μg mL^{-1}). MTT assay was used to detect the viability of cells and the uptake of nanomotors was observed by CLSM. Meantime, HUVECs (normal cells) were treated under the similar procedure to further investigate the targeting ability of the HFLA nanomotors.

Construction of subcutaneous tumor model. BALB/c nude mice (female, 6-8 weeks) were bought from Qinglongshan animal breeding farm, Nanjing, China. Cancer cells (5×10^6 suspended in 150 μL PBS) were subcutaneously injected in the left groin of 6-8-week-old female nude mice. The tumor volumes increase to about 100 mm^3 for about 2 weeks. All animals received humane care in compliance with the *Guide for the Care and Use of Laboratory Animals* published by the National Academy of Sciences, and all experiments procedures and protocols were approved by the Animal Research Committee of Drum Tower Hospital (Nanjing, China, Ethical approval number:2020AE01002).

***In vivo* real-time fluorescence imaging.** DOX and HFLA-DOX nanomotors (200 μL, 200 μg mL^{-1}) were intravenously injected into the MCF-7/ADR tumor-bearing mice. Then, at the specified time intervals (3, 6, 9 h), the mice were anesthetized and scanned using a CRi MaestroTM automated *in vivo* imaging system (C.R. International Inc. USA).

Transvascular and intratumoral penetration. 24-wells transwell insert (polyethylene terephthalate filter, 0.4 μm, Corning) were used to simulate the cells with different layers *in vitro*. To investigate the permeability of nanomotors from vascular to tumor cells. HUVEC cells (5×10^5 mL^{-1}) were seed into a chamber located in the upper 24-well transwell to mimic the vascular barrier to tumor tissue. The HUVECs in the nesting were treated with DOX, HF-

DOX, HF-DOX+L-arginine and HFLA-DOX (200 μg mL^{-1}) for 2 h. The particles that cannot be uptaken by cells was discarded and washed with PBS for three times. The HUVECs insert was replaced with fresh medium and co-incubated with MCF-7/ADR cells in the lower well for 24 h to explore the ability of the samples translocating from the top of HUVECs to bottom tumor cells. The nuclei of cells were stained with Hoechst 33342 (200 μL, 10 μg mL^{-1}, Keygen Bio. Tech.) and the fluorescence images of DOX/nanomotors in HUVECs and MCF-7/ADR cells were observed by CLSM. Besides, to explore the permeability ability of the samples from outer to deeper tumor cells, MCF-7/ADR cells (5×10^5 cells) were seeded into a chamber located in the upper 24-well transwell to simulate the outer tumor cell at tumor tissue. The other treatment procedures were in consistent with the above. The fluorescence intensity was analyzed with Image J software.

Cellular uptake of nanomotors under 2D cellular condition. MCF-7/ADR cells were cultured in petri dish (5×10^4 cells per dish) for 24 h. Cells were cultured with different samples (DOX, HF-DOX, HF-DOX+L-arginine and HFLA-DOX (200 μg mL^{-1})) for 4 h. Subsequently, cell membranes and nuclei were stained with 200 μL DiO (10 μM in DMSO) and 200 μL 4', 6-diamidino-2-phenylindole (DAPI, 0.1μg mL^{-1}) for 15 min, then observed with CLSM. The fluorescence intensity was analyzed with Image J software.

The evaluation of cellular uptake mechanism. The main pathway of nanomotors entering cancer cells was investigated by using different endocytosis inhibitors to treat the cancer cells. MCF-7/ADR cells (5×10^4 cells per dish) were inoculated into the culture dish for 24 h, and MCF-7/ADR were preincubated individually with chlorpromazine (10 μg mL^{-1}), Nystatin (15 μg mL^{-1}), β-cyclodextrin (3 mM), amiloride (13.3 μg mL^{-1}) and 4℃ for 2 h, then the inhibitors were discarded and the inhibitor solution containing the HFLA-DOX (200 μg mL^{-1}) were added to incubate with the cells for another 4 h. To observe the cellular uptake behavior, cells were fixed with 4% formaldehyde solution. Then the cell membranes and nuclei were stained with 200 μL DiO (10 μM in DMSO) and 200 μL DAPI (0.1μg mL^{-1}) for 15 min, and observed with CLSM. The fluorescence intensity was analyzed with Image J software.

Co-localization of nanomotors and lysosomes. MCF-7/ADR cells (1×10^5 cells per dish) in 1 mL medium were seeded in confocal dishes and incubated for 24 h, and then the original medium was removed and DOX, HF-DOX, HF-DOX+L-arginine and HFLA-DOX (200 μg mL^{-1}) were added. After 5 min of incubation, the DMEM was discarded and cells were washed with PBS for three times. Then preheated LysoGreen (1 μM in DMSO, Keygen Bio.Tech.) was incubated with cells for 30 min to make the lysosome labeled green fluorescence. Fluorescence images were taken by CLSM.

Co-localization of nanomotors and Golgi. Golgi tracker green (200 μL, 250 μg mL^{-1} in Hank's/10 mM N-2-hydroxyethylpiperazine-N-2-ethane sulfonic acid (HEPES), Keygen Bio.Tech.) and DAPI were used to make the Golgi labeled green fluorescence and nucleus labeled blue fluorescence after the cells were treated with different samples for 2 h.

Penetration behavior of nanomotors in 3D MTSs. To study the tissue penetration ability *in vitro*, the MTSs with diameter of around 500 μm were sorted out and treated with different samples (DOX, HF-DOX, HF-DOX+L-arginine and HFLA-DOX (200 μg mL^{-1})) for 4 h. All the MTSs were washed for 3 times, then fixed with 4% paraformaldehyde for 15 min and observed under CLSM. The fluorescence images at the different depths of tumor spheroids were obtained using Z-stack scanning from the top to the equatorial plane and the corresponding 3D stacking fluorescence intensity were analyzed by NIS-Elements C software.

Transcellular inhibition experiment in 3D MTSs. The MTSs were pretreated with endocytosis inhibitor Nystain (200 mM in DMSO, ApexBio Technology LLC) for 1 h, then HFLA-DOX nanomotors were added and incubated for 4 h. Besides, another group of MTSs were pretreated with exocytosis inhibitor Exo1 (50 μM in DMSO, ApexBio Technology LLC) for 2 h, then HFLA-DOX (200 μL, 200 μg mL^{-1}) nanomotor was added and treated with the MTSs for 4 h. Fluorescence images were taken by CLSM.

***In vivo* extracellular matrix (ECM) degradation.**

Distribution of NO in the Tumor tissues. Tumor-bearing mice were injected with PBS, HF-DOX, HFLA, HFLA-DOX and HFLA-DOX with oral treated uric acid (10 mg kg^{-1}) intravenously (i. v.). The last group of mice was fed with drinking water containing uric acid (1g kg^{-1}) one day in advance and continued to receive uric acid solution (1g kg^{-1}) by gavage before HFLA-DOX (10 mg kg^{-1}) was injected into tail vein.[6] Six hours after administration, aminomethyl-2',7'-difluorescein, diacetate (DAF-FM DA) (3 mg kg^{-1}, APExBIO, Houston, USA) was injected into the tumor. After 30 min, the mice were killed and the tumor tissue was removed. Tumor tissues were embedded in Tissue-Tek (OCT compound) and immediately frozen in liquid nitrogen. Then, tumor tissue was sectioned (thickness, 10 μm) into slides. Sections were washed with PBS and counterstained with DAPI. All sections were scanned by tissue scanner, pannoramic MIDI (3D HISTECH, Hungary) and processed by pannoramic viewer software. Images are representative sections from three mice per group. The fluorescence intensity was analyzed with Image J software.

Peroxynitrite ($ONOO^-$) detection in the Tumor. Tumor-bearing mice (~100 mm^3) were i. v. injected with PBS, HF-DOX, HFLA, HFLA-DOX and HFLA-DOX+oral treated uric acid (10 mg kg^{-1}). 48 h after injection, the mice were killed, and the tumor were stripped and fixed in 4% formaldehyde, then paraffin embedded and sectioned (thickness, 10 μm). Incubation with the mouse anti-3-nitrotyrosine antibody (Abcam, Ab61392) for 1 h at room temperature, then 3-nitrotyrosine (3-NT) was stained. Hematoxylin is used to make the nucleus purple. All sections were scanned by tissue scanner, pannoramic MIDI (3D HISTECH, Hungary) and processed by pannoramic viewer software. The intensity of 3-NT was analyzed with Densito Quant software.

Expression and Activity of matrix metalloproteinase (MMP). Tumor-bearing mice (~100 mm^3) were i. v. injected with PBS, HF-DOX, HFLA, HFLA-DOX and HFLA-DOX+oral treated uric acid (10 mg kg^{-1}). 48 h after administration, the mice were killed and the tumor was removed. Western blot was used to detect the expression of MMP-1 and MMP-2. The tissue was cut into pieces with scissors, and 400 µL radio immunoprecipitation assay (RIPA) buffer containing phenylmethanesulfonyl fluoride (PMSF) (New Cell&Molecular Biotech. Co. Ltd.) was added to the homogenizer for homogenization. The protein was obtained by centrifugation at 12000 rpm at 4 ℃ for 5 min. The expression of specific protein in 20 µg protein was detected by western blot assay with specific rabbit polyclonal MMP-1 and MMP-2 antibodies (Proteintech Group, Rosemont, IL, USA, 10371-2-AP).

MMP-1 and MMP-2 activity was evaluated using in situ zymography with the EnzChek gelatinase/collagenase assay kit (ThermoFisher Scientific, E12055). The tumor tissue was frozen in liquid nitrogen and embedded in OCT, then cut into 10 µm pieces and placed on the slide. Finally, the enzyme activity in situ was analyzed according to the procedure of the kit. All sections were scanned by tissue scanner, pannoramic MIDI (3D HISTECH, Hungary) and processed by pannoramic viewer software. Images are representative sections from three mice per group. The fluorescence intensity was analyzed with Image J software.

Collagen I assay. Tumor-bearing mice (~100 mm^3) were i. v. injected with PBS, HF-DOX, HFLA, HFLA-DOX and HFLA-DOX+oral treated uric acid (10 mg kg^{-1}). 48 h after administration, the mice were killed and the tumor tissues were harvested. The tumor tissue was fixed in 4% formaldehyde solution and embedded in OCT, then cut into 10 µm sections. The rabbit antimouse collagen I antibody (1:200, Abcam, ab21286) and Alexa Flour 647 donkey antirabbit IgG (Abcam, ab21286) were used for immunofluorescence staining. The nuclei were stained with DAPI. All sections were scanned by tissue scanner, pannoramic MIDI (3D HISTECH, Hungary) and processed by pannoramic viewer software. Images are representative sections from three mice per group. The fluorescence intensity was analyzed with Image J software.

Penetration of nanomotors in tumor. In order to better track the accumulation and penetration of nanomotor in tumor tissue, the tumor-bearing mice (~100 mm^3) were i. v. injected with cy5.5 (Ex 675 nm, Em 694 nm) labeled HF-DOX, cy5.5 labeled HFLA-DOX and cy5.5 labeled HFLA-DOX+uric acid (10 mg kg^{-1}). 24 h after injection, the tumor tissues were harvested and the tumor tissue was frozen into sections. Subsequently, tumor tissue stained with rabbit antimouse CD31 antibody (Abcam, 1:200) and goat antirabbit Aliexa fluor 488 (Servicebio, GB25303, Ex 495 nm, Em 505-550 nm) for whole vessel assay. The nuclei were stained with DAPI (Ex 365 nm, Em 420-470 nm). All sections were scanned by tissue scanner, pannoramic MIDI (3D HISTECH, Hungary) and processed by pannoramic viewer software. Images are representative sections from three mice per group. The fluorescence intensity was analyzed with Image J software.

Therapy ability of the tumor cells under different conditions by the nanomotors.

MTT assay. Cell viability was measured by MTT assay. MCF-7/ADR cells were seeded in 96-wells plate (1×10^4 cells mL^{-1}) and cultured for 24 h. Cell viabilities of MCF-7/ADR treated with 200 μL different samples: L-arginine (100 μg mL^{-1}), HF (100 μg mL^{-1}), HFLA (100 μg mL^{-1}), DOX (50 μg mL^{-1}), HF-DOX (50 μg mL^{-1}), HF-DOX+L-arginine (100 μg mL^{-1}) and HFLA-DOX with different concentrations (50, 100, 150, 200 μg mL^{-1}) were added, and continue to incubate with cells for 24 h. Then MTT reagent (50 μL, 5 mg mL^{-1}) was added to plates and incubated for another 4 h. After that the formazan precipitates were dissolved in DMSO (150 μL), which was transferred to a new 96-well plate and the absorbance was tested with a microplate reader ($OD_{570\ nm}$).

Live/dead cell viability assay. MCF-7/ADR cells were planted in culture dishes with a density of 5×10^4 cells mL^{-1} and treated with 200 μL different samples: L-arginine (100 μg mL^{-1}), HF (100 μg mL^{-1}), HFLA (100 μg mL^{-1}), DOX (50 μg mL^{-1}), HF-DOX (50 μg mL^{-1}), HF-DOX+L-arginine (100 μg mL^{-1}) and HFLA-DOX with different concentrations (50, 100, 150, 200 μg mL^{-1}) for 24 h. The live and dead cells were labeled with Calcein-AM/ propidium iodide (PI) double-stain kit. The cells were incubated with dyeing agent at 37°C for 15 min. And then living cells were stained with green fluorescence by Calcein-AM (2 μM) and the dead cells were stained with red fluorescence by PI (4.5 μM). Fluorescence imaging of cells was observed by CLSM.

***In vitro* MDR reversal mechanism investigation under 2D MCF-7/ADR cellular condition.**

Imaging of intracellular NO release. MCF-7/ADR cells were seeded in 6-well plates and culture dishes at a density of 1×10^5 cells mL^{-1} and cultured for 24 h. Afterward, HF (100 μg mL^{-1}) and HFLA nanomotors (100 μg mL^{-1}) were added into the wells and co-incubated for 4 h. Then, the cells were washed with serum-free medium for several times and dyed with DAF-FM DA (5 μM) for 20 min. Subsequently, fluorescence images were captured by CLSM. After being digested with trypsin, the cells were collected by centrifugation and then suspended in 1 mL medium and the fluorescence intensity was recorded by F-4600 spectrophotometer under excitation at 495 nm and emission at 515 nm.

Measurement of P-gp expression. MCF-7/ADR cells were co-incubated with HF and HFLA nanomotors (100 μg mL^{-1}) for 4 h. Then cells were washed for 3 times with PBS, fixed by 4% paraformaldehyde for 15 min. Subsequently, the P-gp expression of cells was quantified by immunofluorescence stain-test. The first antibody was anti-P glycoprotein antibody (1:200, Abcam ab129450, Cambridge, MA, USA). The second antibody labeled with fluorescein was coralite 488-conjugated affinipure goat anti-rabbit IgG (H+L) (1:300, Proteintech Group, Inc. USA). The nucleus was stained blue by DAPI. Finally, the cells were examined via CLSM (Ex: 493 nm; Em: 519 nm).

Western blots assays. MCF-7/ADR cells with a density of 70-80% confluence were co-incubated with HF and HFLA nanomotors (100 μg mL^{-1}) for 4 h. Then cells were washed with PBS for several times and then lysed by RIPA. The protein concentration of each sample

was measured by Bicinchoninic acid (BCA, Keygen Bio. Tech.). And the proteins of each sample were separated by sodium dodecyl sulfate polyacrylamide and then transferred to a polyvinylidene fluoride (PVDF) membrane. The PVDF membranes were incubated in closed buffer (tris-buffered saline with Tween (TBST) containing 5% skimmed milk) for 1 h. After that, the membranes were cultured with P glycoprotein rabbit polyclonal antibody (1:300 dilutions) and β-actin (1:1000 dilution) at room temperature for 12 h. After being washed with TBST for three times, the blots were incubated with secondary antibody for 30 min and an enhanced chemiluminescence (ECL) was used. The pictures were captured using a chemiluminescent imaging system.

***In vivo* tumor therapy**

Elimination ability of the nanomotors to solid tumors. The mice with MCF-7/ADR tumors (~100 mm^3) were randomly separated into three groups (Control, DOX and HFLA-DOX group) (n=3 per group), which were i. v. injected with DOX or HFLA-DOX nanomotors (10 mg kg^{-1}) at 1, 3, 5, 7, 9, 11, 13 day. The weight of mice and tumor volume were monitored every day for 23 days, then the mice were sacrificed to separate the solid tumor and the major organs of the mice (heart, liver, spleen, lung and kidney). The tumor volume was determined by the following formula: volume = (tumor length) ×(tumor width) ^{2}/2. The separated solid tumor and the organs were fixed with 4% formaldehyde and stained with hematoxylin and eosin (H&E). Besides, the separated solid tumor was stained with anti-Ki67 antibody (1:200, Affinity Biosciences) for immunohistochemistry analysis according to the following procedure. In order to prevent nonspecific binding, the sections were blocked at room temperature for 1 h in blocking buffer containing goat serum. Then anti-Ki67 antibody was added, followed by the addition of appropriate horseradish peroxidase (HRP) conjugated secondary antibody (Proteintech Group, Rosemont, IL, USA) for 30 min at 37℃ and assessed by 3'3-Diaminobenzidine (DAB) coloration. Meantime, the apoptosis in tumor tissue was also detected by TUNE immunofluorescence staining (Beyotime Biotechnology).

Statistical Analysis.

Statistical analyses were performed using SPSSInc. One-way analysis of variance (ANOVA) followed by Tukey post hoc test was used for comparison of three or more groups. Experimental data are mean±s.d. Values were normally distributed, and the variance was similar between compared groups. $P < 0.05$ was considered statistically significant.

Results

Characterizations of the nanomotors.

We quantified L-arginine in HFLA nanomotors using naphthol-diacetyl chromogenic quantification method [5]. The detailed experimental protocol is as follows. 1) Preparation of naphthol-diacetyl chromogenic solution: 1 mL of the solution containing 40 mg mL^{-1} NaOH, 80 mg mL^{-1} methaqualol/n-propanol and 0.5 μL mL^{-1} diacetyln/propanol. 2) Preparation of standard L-arginine solution. L-arginine solutions with concentration gradients of 200, 210, 220, 230, 240, 250, 280, 300 and 320 μg mL^{-1} were prepared, respectively. 3) Detection of L-arginine. 100 μL of standard arginine solution or HFLA (3 mg mL^{-1}) was added to the naphthol-diacetyl chromogenic solution at 30 °C for 15 min. The absorbance of the solution was detected at 570 nm (OD_{570}). The standard curve equation for the concentration of L-arginine and absorbance was Y=0.00109X + 0.16303, R^2=0.999 (Figure S3). The mass fraction of L-arginine in HFLA nanomotors was calculated to be about 10 %. The possible combination mechanism of HF with L-arginine was illustrated in Figure 1 and proven by zeta potential, Fourier transform infrared (FTIR), X-ray photoelectron spectroscopy (XPS) results (Figure 1E, and S4-S6), in which HF displayed cage-like structure with heparin located outside and most of folic acid locating inside the cage.[7] With addition of L-arginine, some of L-arginine will locate outside of HF attracted by negatively charged heparin, and some will be attracted and fixed by the amino groups in folic acid located inside of the cage. The zeta potential of HF is about -51.3 mV (Figure 1E), which changes to -33.9 mV for HFLA. The

decreased surface negative charge may be attributed to the fact that L-arginine located outside HF nanoparticles covering part of the negative charge of HF. Moreover, the structures of HF, HFLA and HFLA-DOX were also characterized by FTIR and XPS spectra (Figure S4-S6), respectively. It can be seen from Figure S4 that the FTIR spectrum of HFLA contains the main peaks existing in both L-arginine and HF. [8, 9] XPS spectra show that the ratio of C-N bond in HF is about 47.3%, indicating that heparin with plenty of C-N groups locates mainly outside of the HF nanoparticles (Figure S5 and S6). Compared with HF, the content of C=N in HFLA increases from 40.4% to 55.7%, indicating that L-arginine may be loaded on both outside and inside of HFLA by electrostatic interaction. In addition, with the loading of DOX, the content of C-N in HFLA-DOX increase by about 5% compared with that in HFLA, which also proves that DOX was successfully loaded into the synthesized nanoparticles.

Targeting ability of the nanomotors to tumor cells.

In order to assess the targeting ability of the HFLA nanomotors to cancer cells, HLA nanomotors without folic acid (reported in our previous work) was introduced in this work.[5] MTT results show that when HFLA and HLA nanomotors are treated with MCF-7/ADR cells, more cells can be inhibited by HFLA nanomotors (about 62% of the cells can be killed) because of their targeting ability, while only about 28% of the cells can be inhibited by HLA nanomotor without targeting ability (Figure S15). When the HFLA nanomotors are treated with HUVECs which expressed less level of folate receptors than cancer cells, about 53% of the HUVECs can be inhibited by HFLA nanomotors, which is lower than that of MCF-7/ADR treated with HFLA nanomotors. CLSM images also display the similar trend as MTT results, which shows that HFLA co-incubated with MCF-7/ADR cells with high expression of folate receptor demonstrate the strongest blue fluorescence signals (Figure S16). These results suggest that folic acid in HFLA nanomotors may facilitate them to target cancer cells with high folic acid receptors expressions.

Transvascular and intratumoral penetration ability of the nanomotors.

The monolayer of HUVECs were seeded in the Transwell insert to mimic the blood vessel barrier at tumor tissue. The HUVECs were incubated with different samples for 2 h, and then the medium was discarded and replaced with fresh medium, and treatment with MCF-7/ADR at the lower chamber for another 24 h to investigate the translation ability from HUVECs to bottom cancer cells.

Moreover, the intratumoral penetration ability of the nanomotors was also investigated by seeding MCF-7/ADR cells in Transwell insert and bottom chamber (Figure 2C, 2D and S18). It can also be concluded that the motion ability of HFLA-DOX nanomotors can greatly promote the transportation between tumor cells. The obvious red fluorescence signals within MCF-7/ADR cells treated with HFLA-DOX nanomotors at the lower chamber imply that drugs can invade the cells efficiently with the motion of nanomotors, while free DOX is rather difficult to penetrate into tumor cells, suggesting that the motion ability of nanomotors can efficiently overcome the vascular barriers and penetrate into deeper tumor tissues through cell-to-cell infiltration.

Cellular uptake and intracellular distribution of nanomotors.

As intracellular distribution is a key factor in the transport and effectiveness of nanomotors, thus, we studied the distribution of nanomotors in cells after they entered the cells. Because many nanoparticles are easy to be swallowed by lysosomes when they enter cells, [10] it is difficult for them to play a role. Therefore, we use lysosome labeling to study the co-localization of lysosomes and nanomotors in cells. The co-localization of nanomotors and lysosomes were detected by using CLSM after being treated for 5 min in order to distinguish

whether HFLA-DOX nanomotors (red color) were transported to lysosomes (LysoTraker, green color) after being internalized in cells. As displayed in Figure 3C and S21, almost all the internalized DOX were co-localized with lysosomes, in which the fluorescence distribution curve of DOX in cells is highly consistent with that of lysosomes. That is to say, the fluorescence signal of DOX displays in the region where lysosomes show strong fluorescence signals, and there is no DOX signal in the regions without lysosomes. In contrast, the fluorescence curve of HFLA-DOX nanomotors in cells shows a low correlation with that of lysosomes. The region without lysosomes still shows a very strong fluorescence signal of the nanomotors, indicating that HFLA-DOX nanomotors can effectively escape the phagocytosis of lysosomes. Such fast and efficient drug penetration revealed that the nanomotors proposed in this work can readily overcome the main biological barriers of cancer cells, thus offering much higher drug delivery ability.
Further, we also co-localized the nanomotors with Golgi (main organelle involved in cell transport) and nucleus (main organelle that DOX kills cancer cells by destroying the tertiary structure of DNA).[11, 12] As shown in Figure 3D and S22, it is difficult for DOX alone to reach the nucleus or Golgi after incubation for 2 h. From the corresponding fluorescence spectra, the peak position of DOX does not coincide with that of Golgi and nucleus. For nanomotor group, the fluorescence coincidence of blue nucleus, green Golgi and red nanomotors is higher. The fluorescence spectra of red nanomotors are well in consistent with that of nucleus and Golgi, implying that the nanomotors partly distribute in the nucleus and part in Golgi. The nanomotors distributed on the Golgi can be effectively transported outside the cells (exocytosis), and then use the movement of the nanomotor itself to promote its endocytosis and exocytosis by the next cell, effectively completing cell-to-cell transmission.

The fact that nanomotor can effectively escape the phagocytosis of macrophages was proved as follows. In this work, the nanomotor we proposed has the ability to target the folate receptor overexpressed on the surface of cancer cells, so it can be effectively taken up by cancer cells. In addition, because the cancer cell environment has a notably higher hydrogen peroxide concentration than normal cells such as macrophages[13]. It is the higher concentration of ROS and NOS in the cancer cell environment that allows the nanomotor to move (Figure S23, Movie S7), while the nanomotor cannot move in the macrophage environment (Figure S24, Movie S8), which may be the main reason why macrophages take up HFLA-DOX less efficiently. To verify this hypothesis, we measured the uptake efficiency of macrophages for HF-DOX+L-arginine and HFLA-DOX, which displayed that the cellular uptake efficiencies for the above two groups were less than 8%, further validating the low phagocytosis efficiency of the macrophages to the nanomotor proposed in this work, and also verified the above speculation (Figure S25).

The above results indicated that the enhancement of cellular endocytosis by nanomotors may be owing to their targeting and motion ability in cancer cells, while the inhibition of macrophage phagocytosis may be attributed to lacking targeting and motion ability of the nanomotors in macrophages.

Penetration behavior of nanomotors in 3D MTSs.
As shown in Figure 4A, only little red fluorescence representing DOX can be detected in MCF-7/ADR MTSs across each equatorial section for free DOX sample, in which the penetration depth is about 60 μm with rather weak fluorescence signals. In contrast, the penetration depth can be increased to 120 μm for HFLA-DOX nanomotors and much stronger fluorescence signal was observed. Furthermore, the fluorescence intensity profiles across the 120th μm section of the MTSs were summarized in Figure 4B. From the fluorescence intensity profiles of this cross section, it can be seen that the fluorescence intensity of DOX group is very weak (less than 100) around 100 μm. For HFLA-DOX nanomotors, the fluorescence

spectrum shows that the peak intensity (around 3000) is still very high at about 100 μm. For HF-DOX+L-arginine group without motion ability, the penetration depth and fluorescence intensity are much lower than that of nanomotor group. These comparative data show that the motion behavior of nanomotors can greatly promote the penetration process of drugs in MTSs. In particular, the endocytosis inhibitor Nystain and exocytosis inhibitor Exo1 were used to treat 3D MTSs to study the permeation behavior of nanomotors in cell spheres.[14] As shown in Figure 4C and 4D, the MTSs without inhibitor display good wettability after co-culturing with nanomotors for a period of time (red and blue signal represent the color of DOX and nanomotors respectively, and their high coincidence indicates that both red and blue signal can represent nanomotors). However, after co-culturing with endocytosis inhibitor Nystain, the signal of nanomotors that penetrate into the deep part of the MTSs decreases greatly, indicating that it is difficult for the nanomotors to enter the cell when the endocytosis behavior is inhibited. Similar results can be found on the MTSs treated by exocytosis inhibitor Exo1, which suggests that the transport of nanomotors between cells was greatly suppressed after inhibition of exocytosis. These results confirm that the nanomotors are dependent on cell endocytosis to enter the cell, and through Golgi transport (exocytosis) to achieve its transfer between cells.

***In vivo* ECM degradation.**
Different from the control group set up when studying the interaction mechanism of nanomotors with cells, where there are HFLA-DOX nanomotors with NO release ability and motion ability and HF-DOX + L-arginine with NO only but not motion ability was set as the focus of comparison (motion ability was set as a variable) to determine the effect of nanomotor movement on their interaction with cells. In this part, HFLA-DOX nanomotors, which has both NO release ability and motion ability, and HFLA-DOX nanomotors +uric acid, which only has motion ability and cannot produce $ONOO^-$ to degrade ECM, were used ($ONOO^-$ produced by NO was set to a variable) to determine the effectiveness of $ONOO^-$ produced by NO in the above process.

Therapy ability of the nanomotors under different conditions.

Reversal of MDR of cancer cells. As depicted in Figure 6A and S32, control MCF-7/ADR cells display very weak fluorescence signals, while the cells treated with HFLA emit much stronger fluorescence signals, implying that enormous amount of NO can be generated by nanomotors under cellular condition. The corresponding fluorescence spectra and intensity comparison were also summarized in Figure 6B, in which HFLA display much higher fluorescence intensity than that in control group. Then, the effectiveness of the produced NO by HFLA nanomotors as P-gp inhibitors was measured by using western blot assay. From Figure S33, compared with control group, it is obvious that the P-gp expression was remarkably downregulated for cells treated with HFLA nanomotors. Meantime, the inhibition of P-gp expression was also confirmed by immunofluorescence staining (Figure 6C, 6D, and S34), which displays significant decreased fluorescence signal of P-gp for cells treated with HFLA nanomotors. These results indicate that the P-gp protein levels of the MCF-7/ADR cells decrease a lot by the released NO in the cells.

***In vitro* cytotoxicity of the nanomotors.** As can be seen in Figure S35 and table S1, the IC50 value of MCF-7/ADR cells used in this work is much higher than 100 μM, and DOX displays low anticancer ability on MCF-7/ADR cells. As shown in Figure 6E, HF and HFLA nanomotors display certain anticancer ability for that the cell viability of cells decreases to about 86% and 79% after treated with these samples for 24 h. Meantime, DOX also displays certain cytotoxicity to MCF-7/ADR cells. HFLA-DOX shows the best anticancer performance

to MCF-7/ADR cells, and only 50% of the cells maintain their viability, which further decrease with the increasing concentration of HFLA-DOX nanomotors.

Discussion

In this work, we demonstrate that the NO-driven nanomotors can achieve targeted recognition, penetration of blood vessels, intercellular penetration, special intracellular distribution (escaping from lysosomes and accumulating in Golgi and nucleus), 3D MTSs penetration, degradation of tumor ECM, and reverse the MDR of tumors based on the motion ability and sustained NO release performance, which is also the key feature different from current reported NO nanocarriers (Table S1). Due to the lack of a synergistic effect of self-driving ability on penetration, most of the current NO nanocarriers listed in Table S1 can only perform a few parts of the above functions (generally less than three of them).

We first characterize the interaction of nanomotors with cells in detail to assess the important role of their motor effects in this process at the cellular level. Specifically, HF in nanomotors can target tumor tissue (Figure S15 and S16). The guanidine group in L-arginine can form double ligand hydrogen bonds with phosphate and carboxyl groups on the surface of tumor cell membrane,[15, 16] inducing and promoting endocytosis process, which can be confirmed by the pathway of the nanomotor into the cell, that is, the nanomotor relies on the clathrin and caveolin-mediated pathway to enter the cell (Figure 3A and 3B). The movement of nanomotors can further accelerate the process of endocytosis and escaping from lysosome (Figure 3C). Apart from locating on the nucleus of tumor cells, part of the nanomotors can also concentrate on the Golgi, which can transport part of the nanomotors through exocytosis into the next tumor cell (Figure 3D). The released anti-cancer drug DOX near the nucleus kills cancer cells and the movement of the nanomotors can also promote the exocytosis process. By comparing the results of the interaction between HFLA-DOX nanomotors and HF-DOX + L-arginine, groups, it is determined that the motor ability of the nanomotor promotes cellular uptake, intracellular distribution and intercellular transport. For example, the quantitative fluorescence data of the former is 1.5 times that of the latter when detecting the performance of nanomotor penetration into blood vessels, the fluorescence intensity of intercellular penetration of the former is 1.5 times that of the latter, and the fluorescence intensity of the result of cellular uptake is 1.4 times that of the latter (Figure 2). HFLA-DOX nanomotors can effectively complete lysosome escape and accumulation in the nucleus and Golgi, while HF-DOX+L-arginine group without motion ability cannot effectively achieve the above purpose (Figure 3). At the same time, the 3D MTS was used to confirm the promotion effect of the movement effect of the nanomotor on the transport between cells. The penetration depth of the former (approximately 120 μm) is significantly better than the latter (approximately 80 μm) (Figure 4).

Then we use *in vivo* experiments to characterize the degradation of the tumor ECM by the continuous production of NO by the nanomotor, and the details of NO production, $ONOO^-$ production, MMP (MMP-1 and MMP-2) activity, and collagen degradation after the NO-driven nanomotor entered the tumor during the process were fully characterized. By comparing the above results of HFLA-DOX nanomotors group with HFLA-DOX+uric acid group, the key role of the formed $ONOO^-$ in the degradation of the collagen in tumor ECM was confirmed (Figure 5).

Finally, *in vivo* tumor tissue penetration experiments were applied to confirm the synergistic effect of the motion effect of nanomotors and the versatility of NO in deep tissue penetration. Observing the tumor penetration results of the HFLA-DOX+uric acid group (with only motion ability), which can still release NO to promote movement but the generated $ONOO^-$ will be eliminated and cannot degrade ECM, it can be found that its penetration effect is significantly better than that of the pure HF-DOX group. The relative fluorescence intensity is 2 times that of HF-DOX group, indicating that motion ability of the nanomotors can

promote the penetration of drugs in tumor tissues to a certain extent. The HFLA-DOX nanomotors (with both motion ability and $ONOO^-$ formation ability) shows the best penetration effect, thus reflecting the synergistic effect between the motion ability of the nanomotor and the produced $ONOO^-$ degradability of ECM, which greatly promotes its role in deep penetration inside tumor tissue.

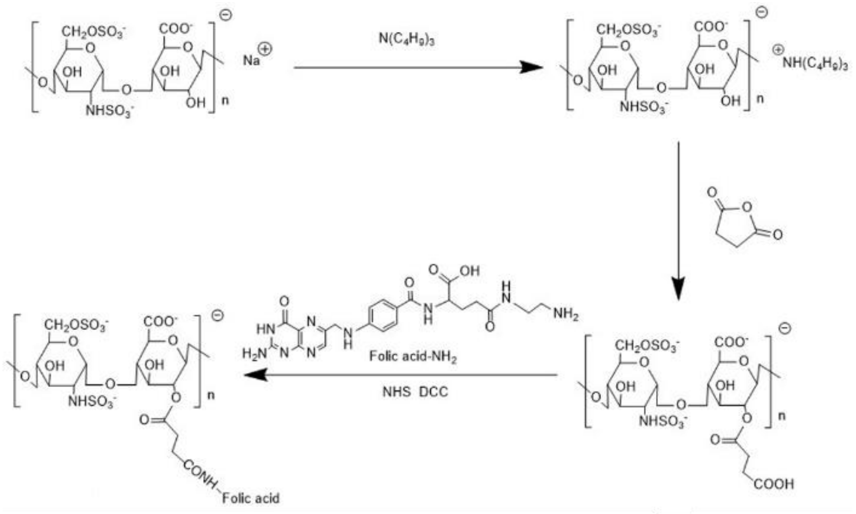

Figure S1. The synthetic procedure of HF composite.

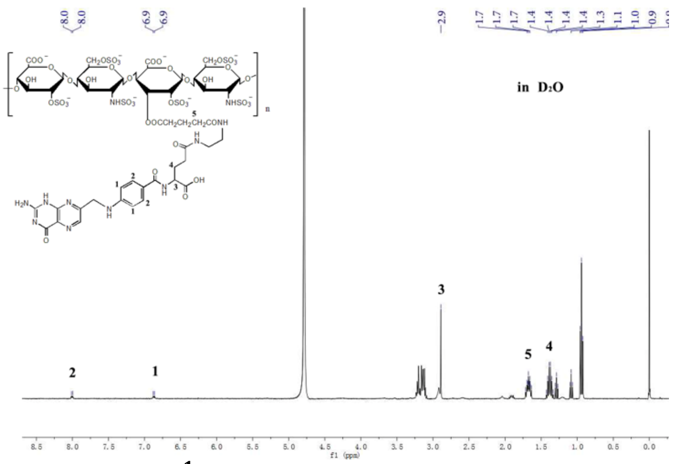

Figure S2. ^{1}H NMR spectrum of HF in D_2O.

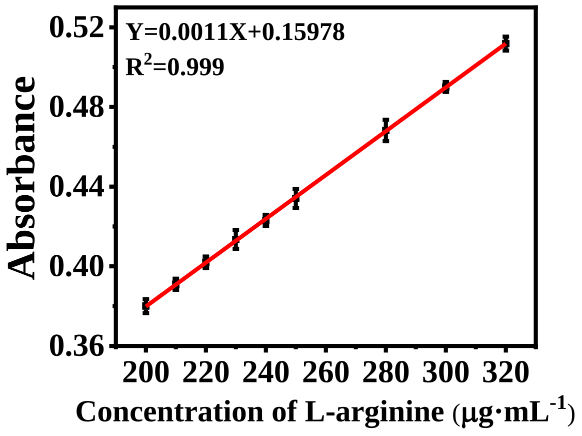

Figure S3. Standard curve of L-arginine concentration versus absorbance. Experimental data are mean ± s.d. of samples in a representative experiment (n = 5).

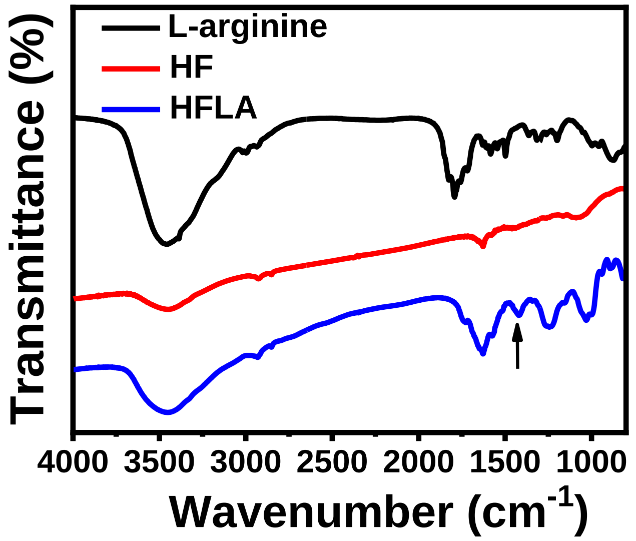

Figure S4. FTIR spectra characterization. FTIR spectra of L-arginine, HF and HFLA nanomotors.

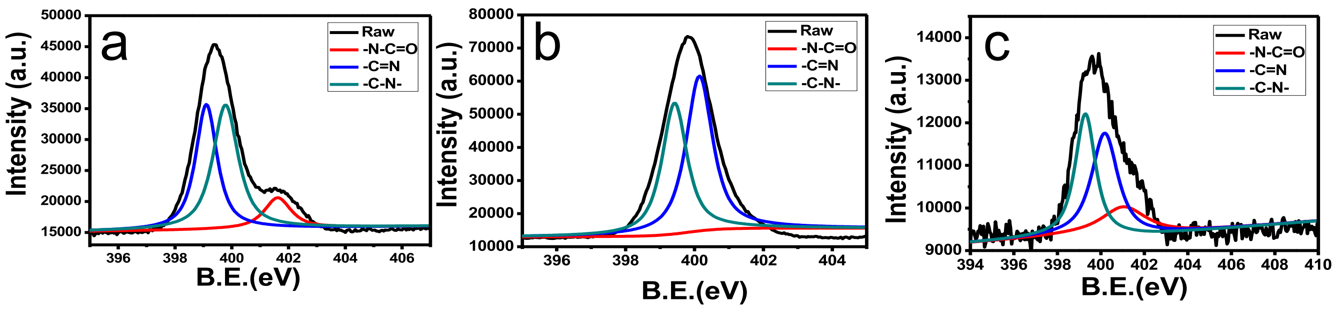

Figure S5. N1s spectra characterization. N1s spectra of (a) HF, (b) HFLA nanomotors and (c) HFLA-DOX nanomotors.

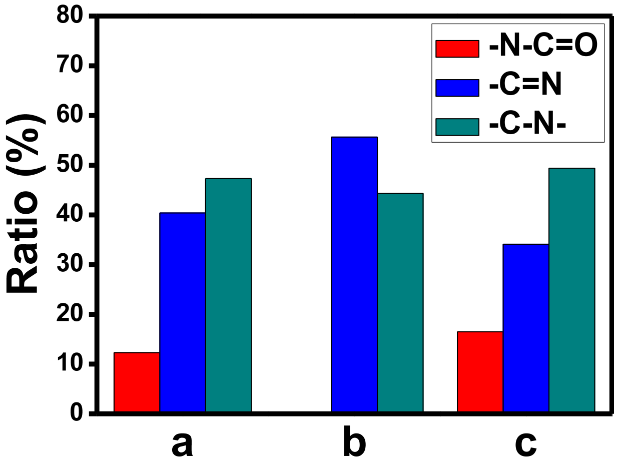

Figure S6. N1s ratio characterization. N1s ratio of (a) HF, (b) HFLA nanomotors and (c) HFLA-DOX nanomotors.

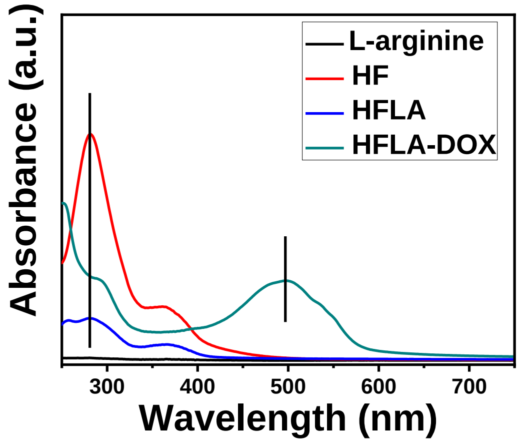

Figure S7. UV-vis spectra characterization. UV-vis spectra of L-arginine, HF, HFLA nanomotors, and HFLA-DOX nanomotors.

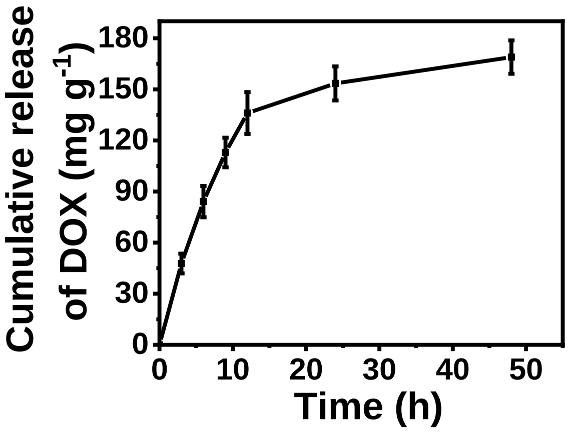

Figure S8. DOX release characterization. Cumulative DOX release profiles of HFLA-DOX nanomotors under PBS solution at 37 ℃. Experimental data are mean±s.d. of samples in a representative experiment (n = 3).

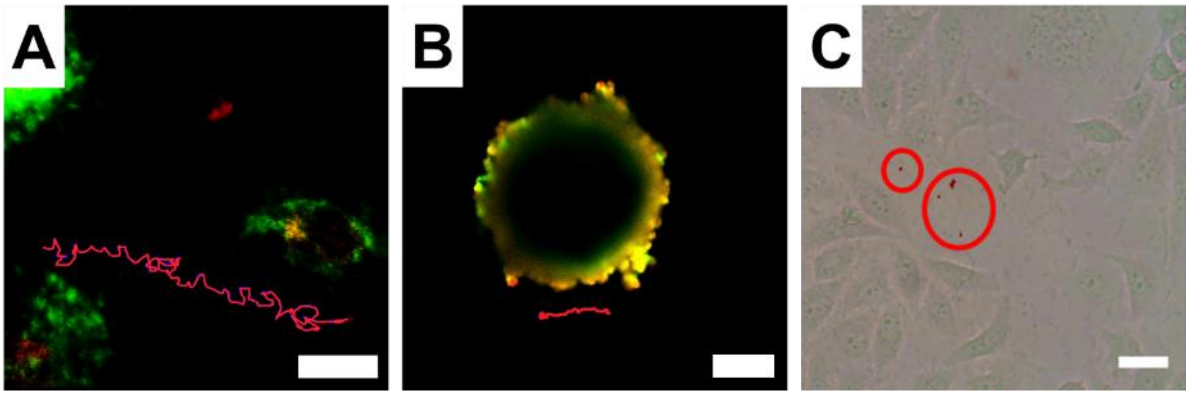

Figure S9. Time-lapse images (movie S1 and S2) displaying the tracking trajectories of HFLA-DOX nanomotors under (A) MCF-7/ADR (10 s, play at 32× speed) (Scale bar: 10 μm) and (B) 3D MTSs solution (6 s, play at 32× speed) (Scale bar: 100 μm) captured by CLSM; and (C) Trajectory of HFLA nanomotors in a cellular environment treated with total NOS inhibitors. (Scale bar: 50 μm， Movie S3, 30 s).

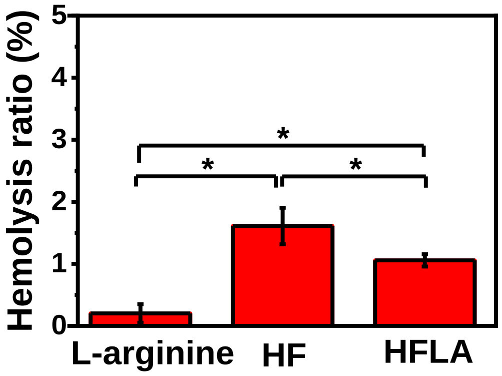

Figure S10. Hemolysis ratios test. Hemolysis ratios of L-arginine, HF, and HFLA nanomotors. Asterisk (*) denotes statistical significance between bars (*$p < 0.05$) using one-way ANOVA analysis. Experimental data are mean±s.d. of samples in a representative experiment (n = 5).

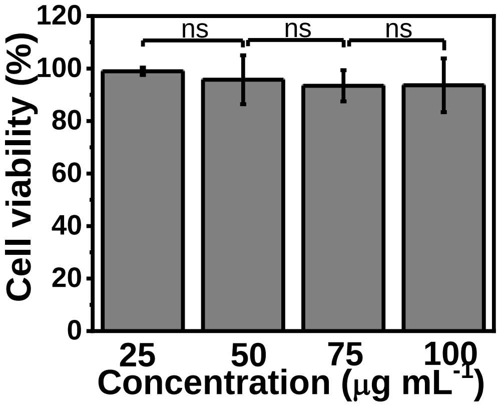

Figure S11. Cell of RAW264.7 viability test. MTT results of HFLA nanomotors cocultured with RAW264.7 under different concentrations: 25 μg mL^{-1}, 50 μg mL^{-1}, 75 μg mL^{-1}, and 100 μg mL^{-1}. Asterisk (ns) denotes statistical significance between bars (ns >0.05) using one-way ANOVA analysis. Experimental data are mean±s.d. of samples in a representative experiment (n = 5).

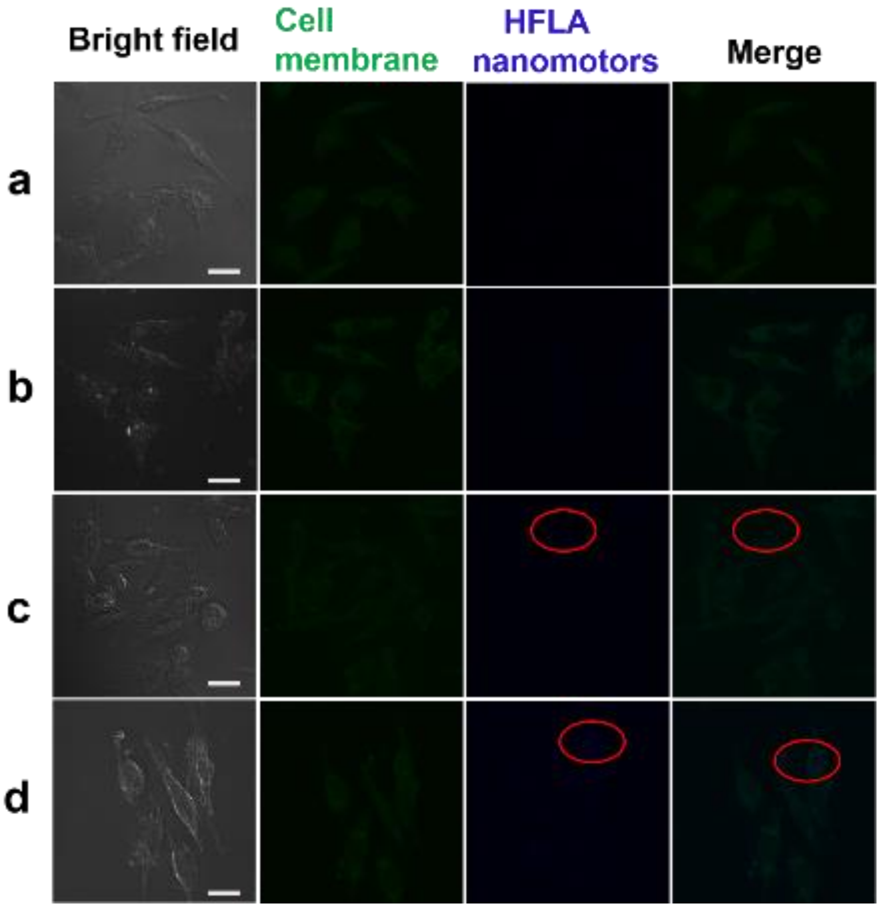

Figure S12. The cellular uptake of HFLA nanomotors test by macrophage. Confocal laser scanning microscopy images of the cellular uptake of HFLA nanomotors with different amounts by RAW264.7 cells for 1 h: (a) 25 μg mL^{-1}, (b) 50 μg mL^{-1}, (c) 75 μg mL^{-1}, and (d) 100 μg mL^{-1} (Scale bar=20 μm) (Cell membrane: green; HFLA nanomotors: Blue).

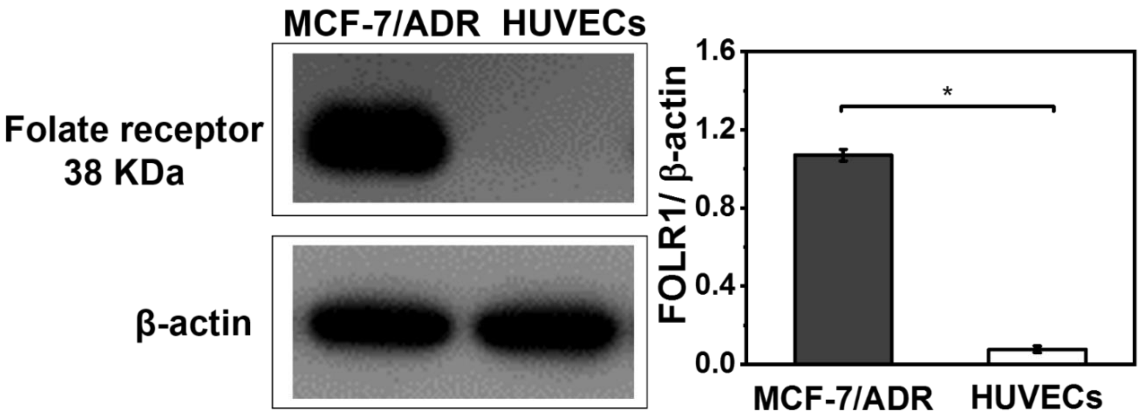

Figure S13. Western blot analysis of the level of folate receptor (MW=38 KDa) in MCF-7/ADR and HUVECs cells. Asterisk (*) denotes statistical significance between bars (*$p < 0.05$) using one-way ANOVA analysis. Experimental data are mean±s.d. of samples in a representative experiment (n = 3).

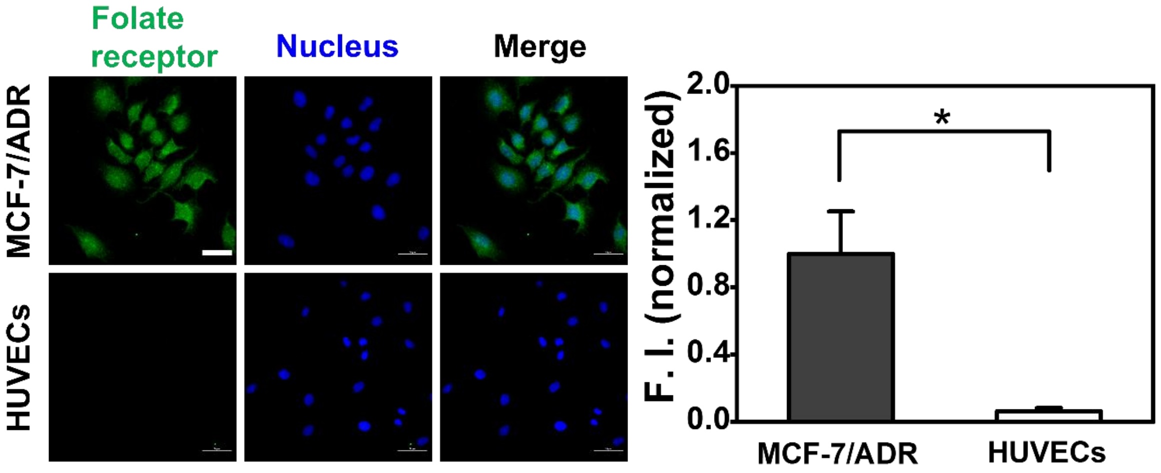

Figure S14. Immunofluorescence analysis of the level of folate receptor protein in MCF-7/ADR and HUVECs cells and its corresponding fluorescent intensity (Scale bar: 50 μm); Asterisk (*) denotes statistical significance between bars (*$p < 0.05$) using one-way ANOVA analysis. Experimental data are mean±s.d. of samples in a representative experiment (n = 3).

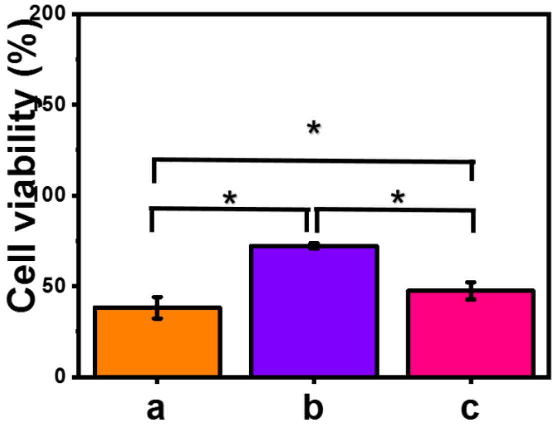

Figure S15. Targeting ability test. Cell viabilities of cells cultured under different conditions: (a) MCF-7/ADR + HFLA nanomotors, (b) MCF-7/ADR + HLA nanomotors, and (c) HUVECs + HFLA nanomotors for 24 h. Asterisk (*) denotes statistical significance between bars (*$p < 0.05$) using one-way ANOVA analysis. Experimental data are mean±s.d. of samples in a representative experiment (n = 3).

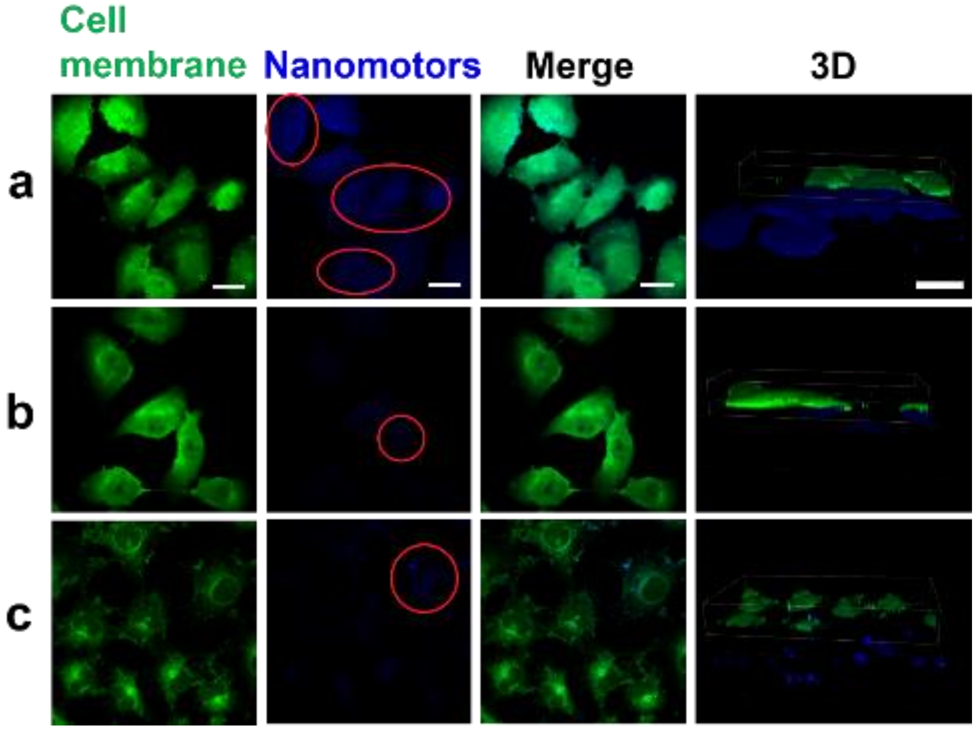

Figure S16. Targeting ability test by cellular uptake characterization. The uptake of cells cultured under different conditions: (a) MCF-7/ADR + HFLA nanomotors, (b) MCF-7/ADR + HLA nanomotors, (c) HUVECs + HFLA nanomotors for 4 h, and (d) their corresponding snapshots of 3D rendered Movies made from a stack of confocal images. (Blue: nanomotors was circled in red; Green: cell membrane, scale bar: 20 μm, movie S4).

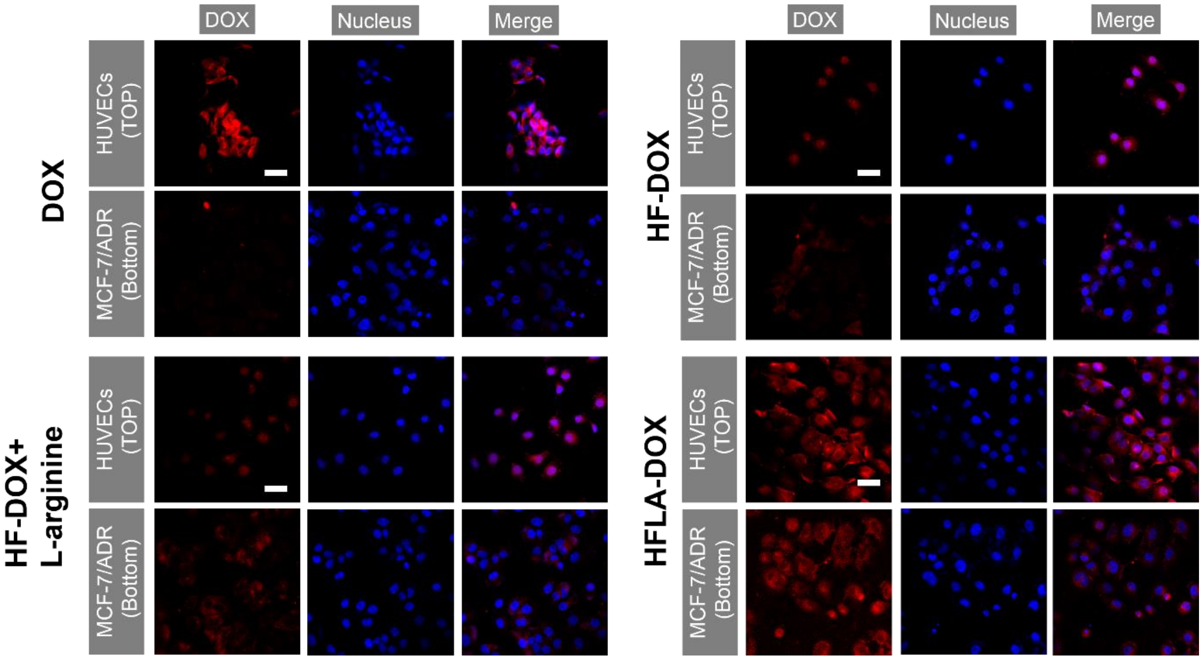

Figure S17. Penetration ability test under 2D condition. CLSM images for *in vitro* experimental models of transvascular extravasation penetration: DOX, HF-DOX, HF-DOX+L-arginine, and HFLA-DOX nanomotors, (Blue: nucleus; Red: nanomotors; Scale bar=50 μm).

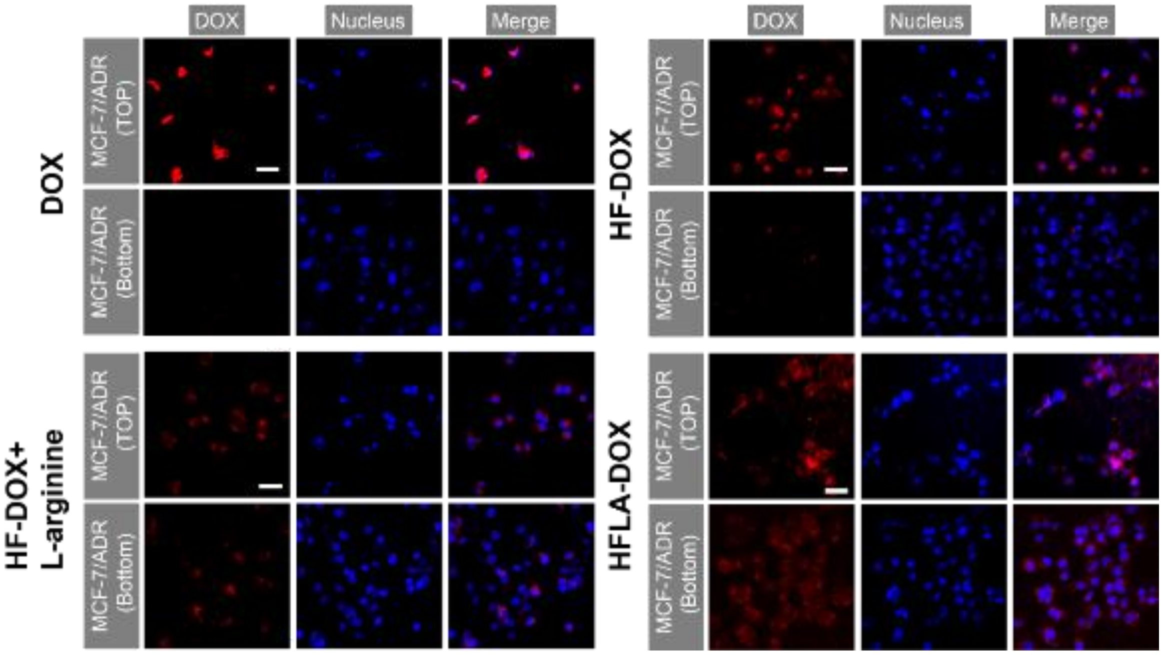

Figure S18. Penetration ability test under 2D condition. CLSM images for *in vitro* experimental models of intratumoral penetration: DOX, HF-DOX, HF-DOX+L-arginine, and HFLA-DOX nanomotors, (Blue: nucleus; Red: nanomotors; Scale bar: 50 μm).

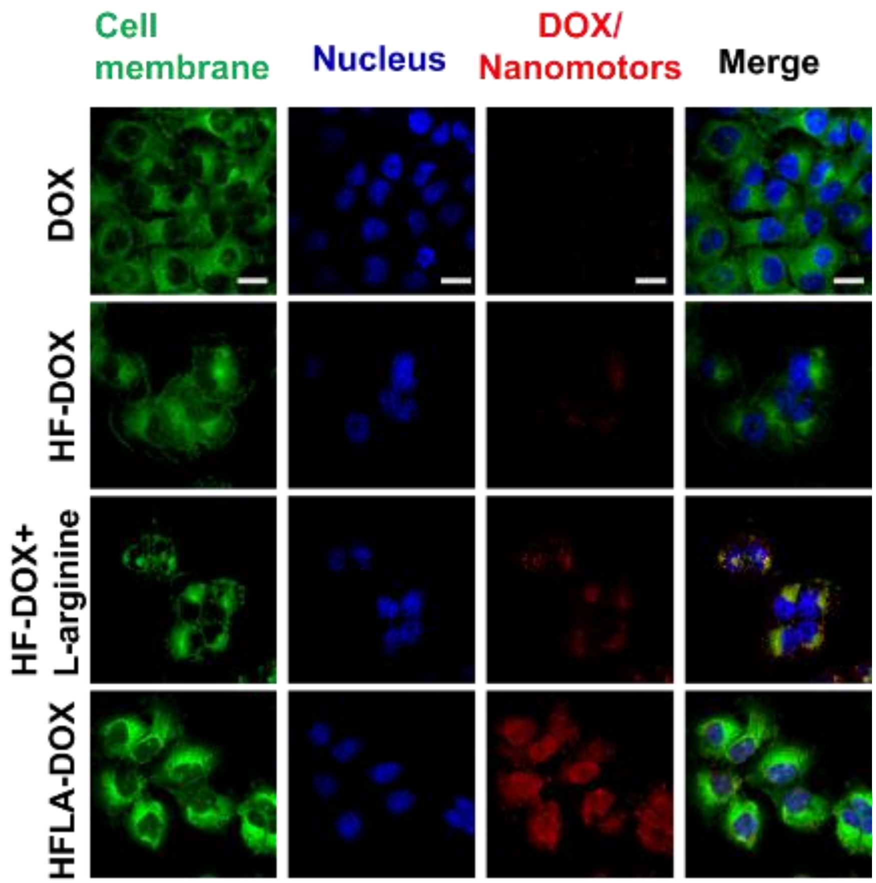

Figure S19. The cellular uptake was in 2D cellular condition. CLSM images of MCF-7/ADR cells after uptaking the nanomotors under different conditions: DOX, HF-DOX, HF-DOX+L-arginine, and HFLA-DOX (Green: cell membrane; Blue: nucleus; Red: nanomotors; Scale bar: 20 μm, Movie S5).

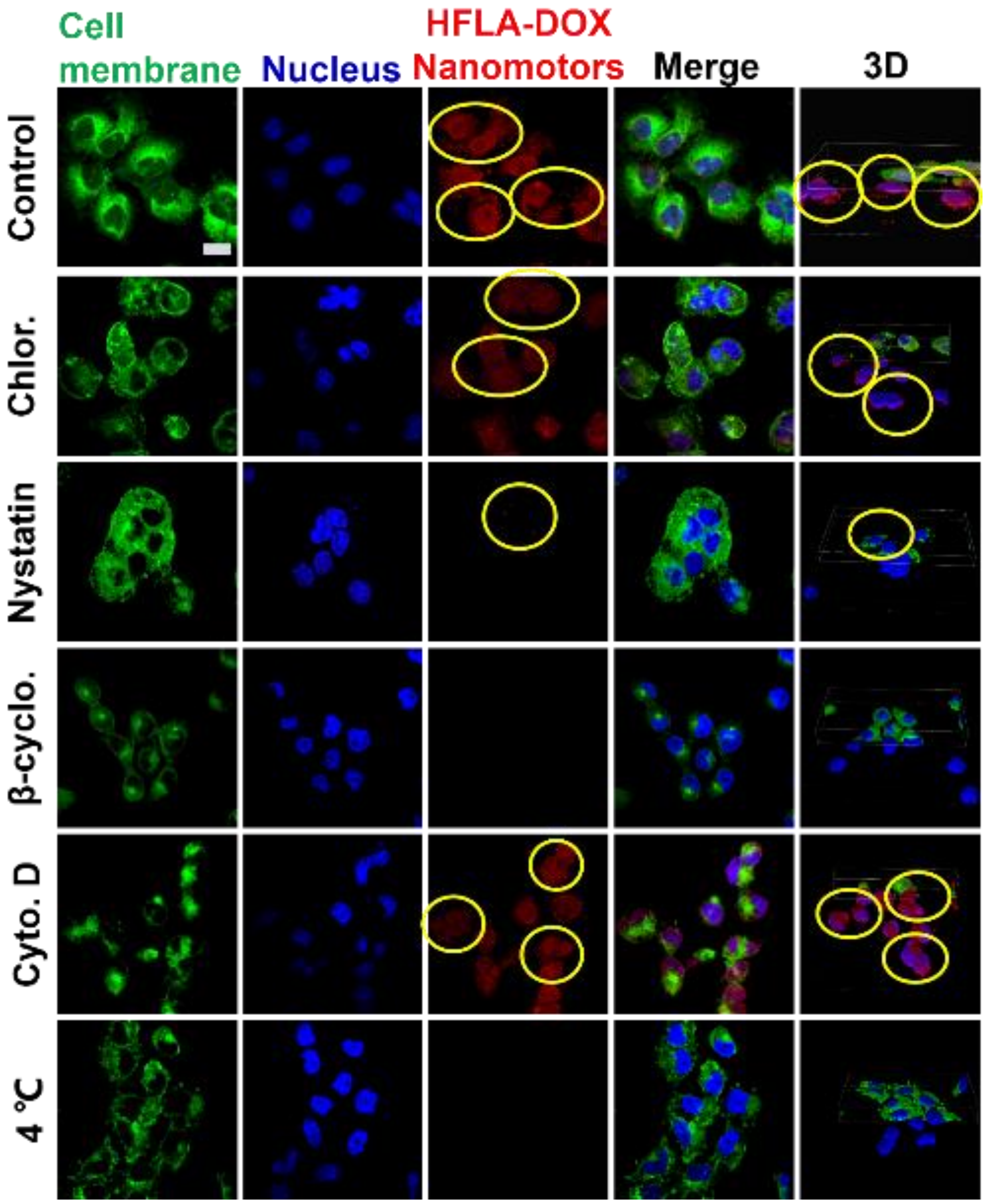

Figure S20. Detection of the main pathway of nanomotors entering cancer cells. CLSM images of MCF-7/ADR cells after being incubated with nanomotors for 4 h at the absent of (control) or presence of different endocytic inhibitors (chlorpromazine (chlor.), Nystatin, β-cyclodextrin (β-cyclo.), cytochalasin D (cyto. D), e: 4 ℃); (Green: cell membrane; Blue: nucleus; Red: nanomotors; Scale bar: 20 μm, Movie S6).

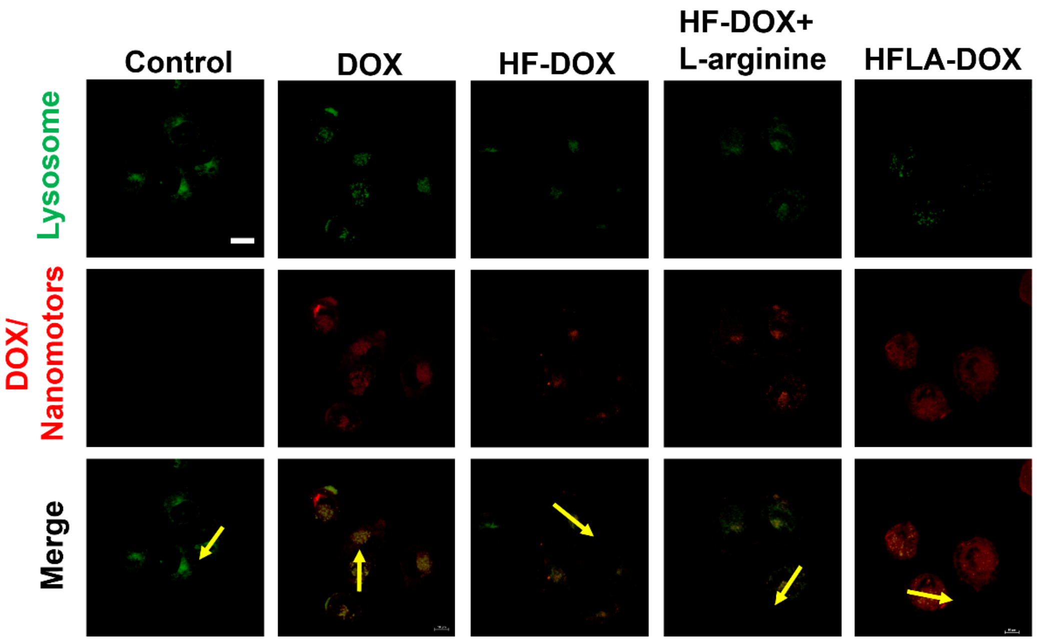

Figure S21. The distribution of nanomotors. Colocalization of control, DOX, HF-DOX, HF-DOX+L-arginine, and HFLA-DOX nanomotors (red) with lysosome (green, 5 min) after being cultured with MCF-7/ADR cells (Scale bar: 20 μm).

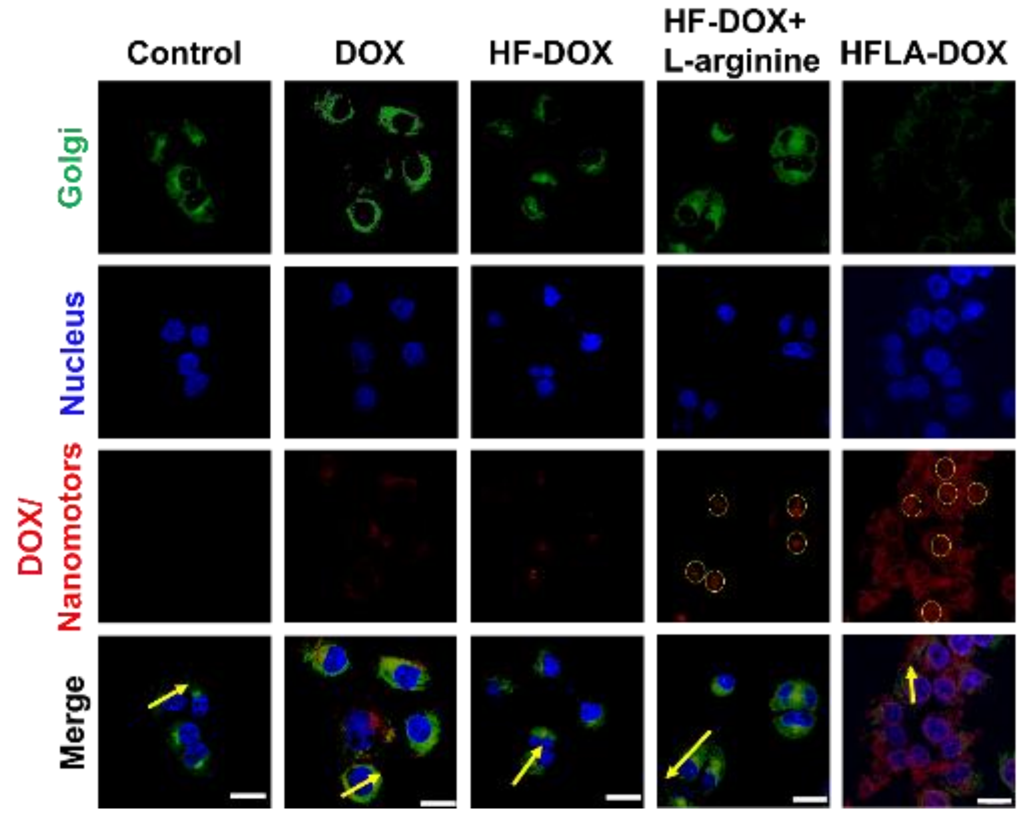

Figure S22. The distribution of nanomotors. Colocalization of control, DOX, HF-DOX, HF-DOX+L-arginine, and HFLA-DOX nanomotors (red) with nucleus (blue)/Golgi (green) (2 h) after being cultured with MCF-7/ADR cells (Scale bar: 20 μm).

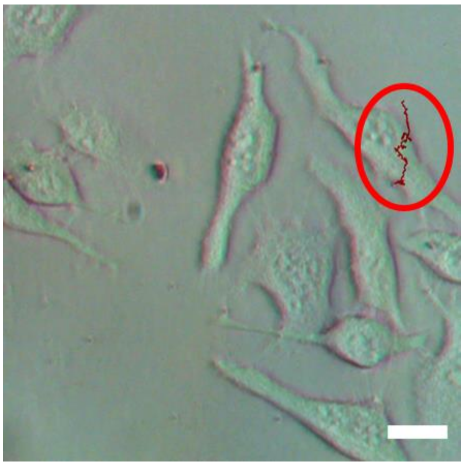

Figure S23. Trajectory of HFLA-DOX nanomotors in MCF-7/ADR cellular environment (Scale bar: 50 μm, movie S7, 30 s).

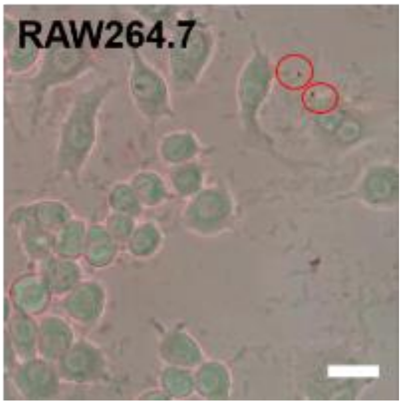

Figure S24. Motion behavior of HFLA-DOX nanomotors in RAW246.7 cellular environments. (Movie S8, 30 s).

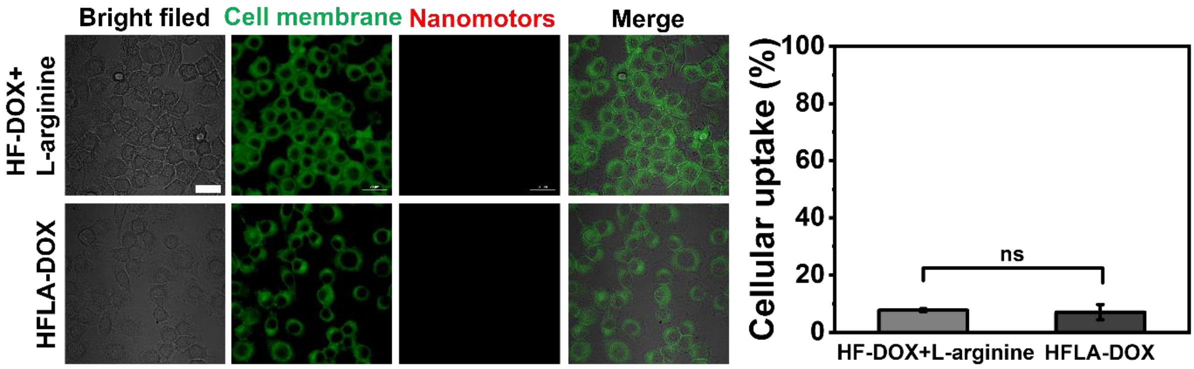

Figure S25. CLSM images and cellular uptake efficiency of RAW264.7 cells after uptaking the nanomotors under HF-DOX+L-arginine, and HFLA-DOX nanomotors (Scale bar: 20 μm). Asterisk (ns) denotes statistical significance between bars (ns>0.05) using one-way ANOVA analysis. Experimental data are mean±s.d. of samples in a representative experiment (n = 5).

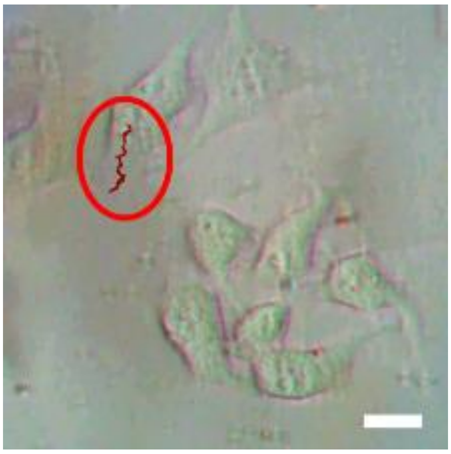

Figure S26. Trajectory of HFLA-DOX nanomotors + uric acid in MCF-7/ADR cellular environment (Scale bar: 50 μm, movie S9, 30 s).

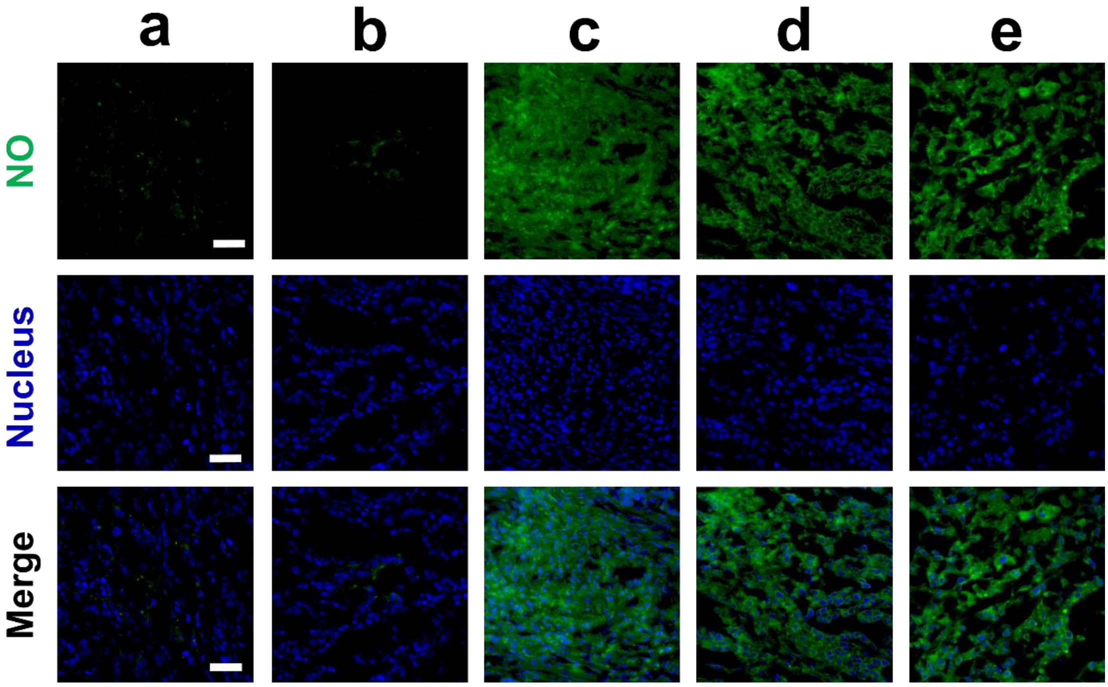

Figure S27. Detection of NO in tumor. Fluorescence images of NO under different condition: (a) PBS, (b) HF-DOX, (c) HFLA nanomotors, (d) HFLA-DOX nanomotors, and (e) HFLA-DOX nanomotors +uric acid in tumor tissues using DAF-FM DA fluorescent probe (Scale bar: 50 μm)

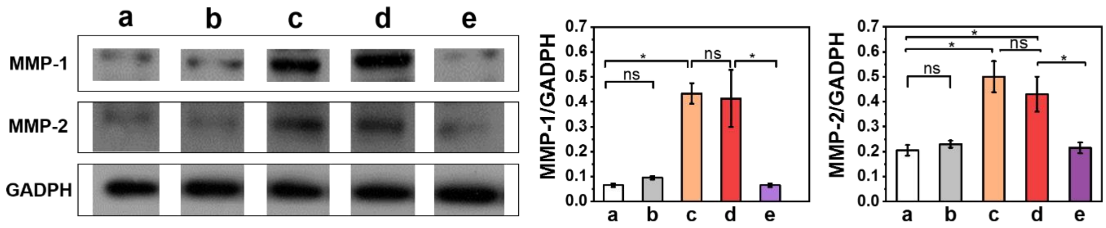

Figure S28. Western blot assay. Western blot assay showed the expression of MMP-1 and MMP-2 treated with (a) PBS, (b) HF-DOX, (c) HFLA, (d) HFLA-DOX nanomotors, (e) HFLA-DOX nanomotors +uric acid and corresponding statistical assay of MMP-1 and MMP-2 contents. Asterisk (*) denotes statistical significance between bars (*$p < 0.05$) using one-way ANOVA analysis. Experimental data are mean±s.d. of samples in a representative experiment (n = 3).

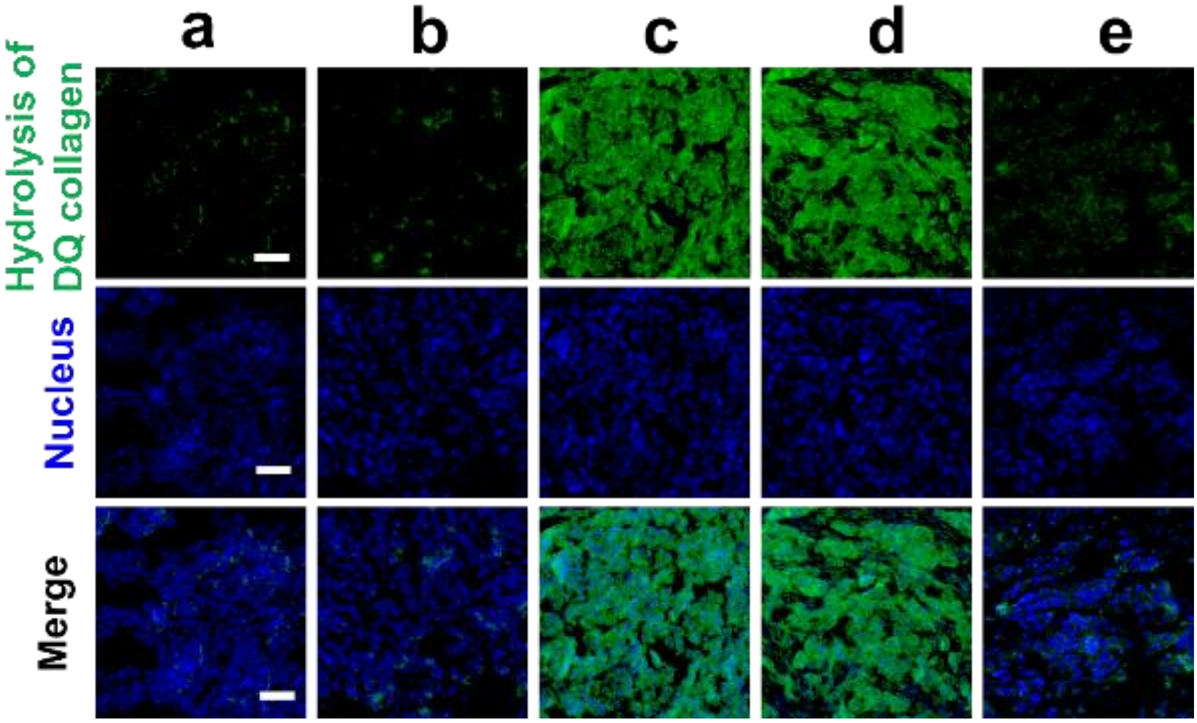

Figure S29. In situ zymography assay of tumor cryosections (10 μm). Green, fluorescence signal resulted from the hydrolysis of DQ collagen (quenched fluorescence due to heavily conjugated fluorescein) by collagen enzymes; (a) PBS, (b) HF-DOX, (c) HFLA nanomotors, (d) HFLA-DOX nanomotors, and (e) HFLA-DOX nanomotors +uric acid (Blue, nucleus; Scale bar: 50 μm).

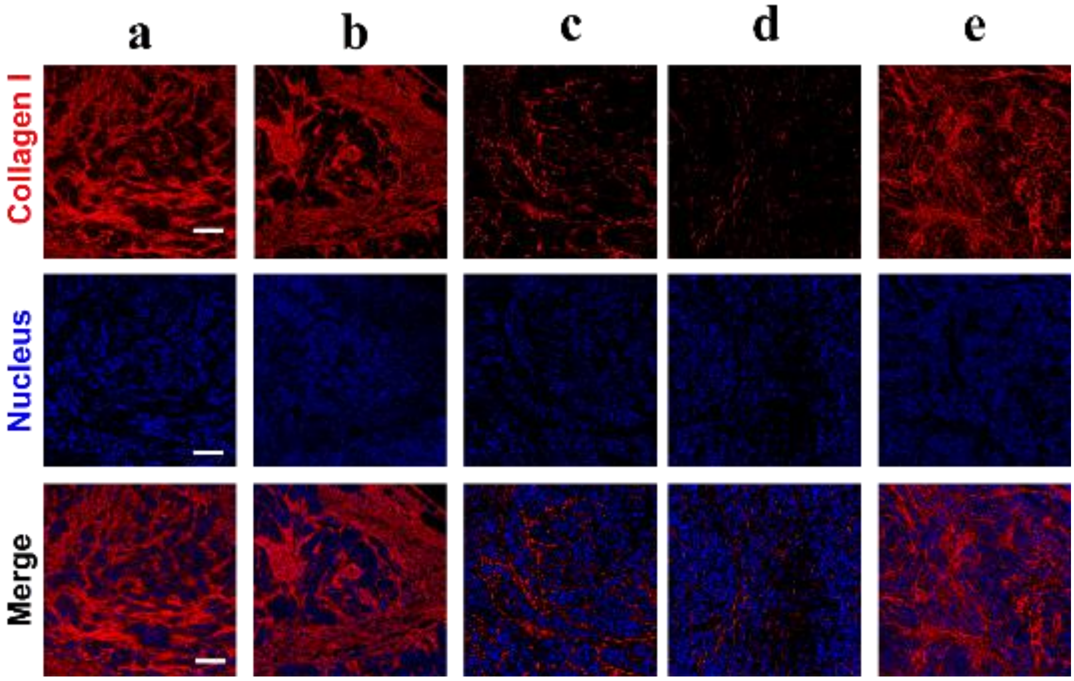

Figure S30. Immunofluorescent staining of Collagen I in tumors. Samples for (a) PBS, (b) HF-DOX, (c) HFLA nanomotors, (d) HFLA-DOX nanomotors, and (e) HFLA-DOX nanomotors +uric acid (Red color: collagen I; blue color: nucleus; Scale bar: 50 μm).

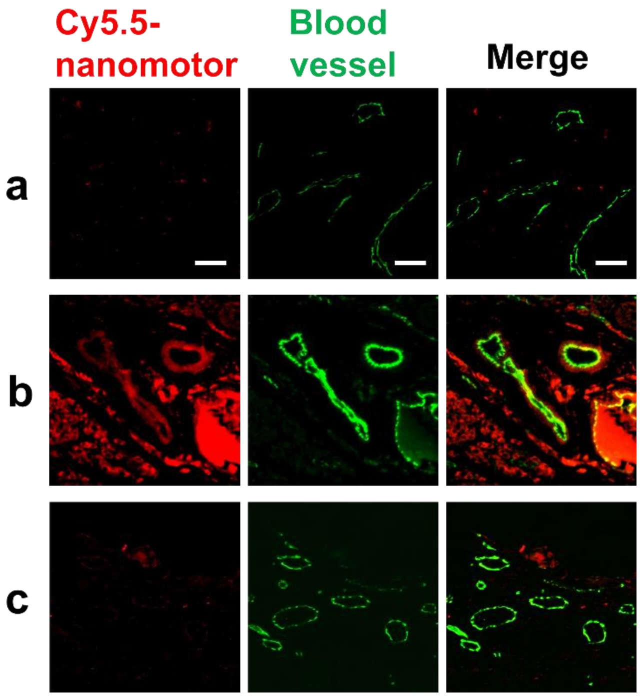

Figure S31. The penetration of the nanomotor in the tumor blood vessels. Immunofluorescence images displaying the distribution of cy5.5 stained samples (red color) in tumor (blood vessels were stained with CD31 antibody, green color), samples for (a) cy5.5 labeled HF-DOX, (b) cy5.5 labeled HFLA-DOX nanomotors, and (c) cy5.5 labeled HFLA-DOX nanomotors +uric acid (Scale bar: 50 μm).

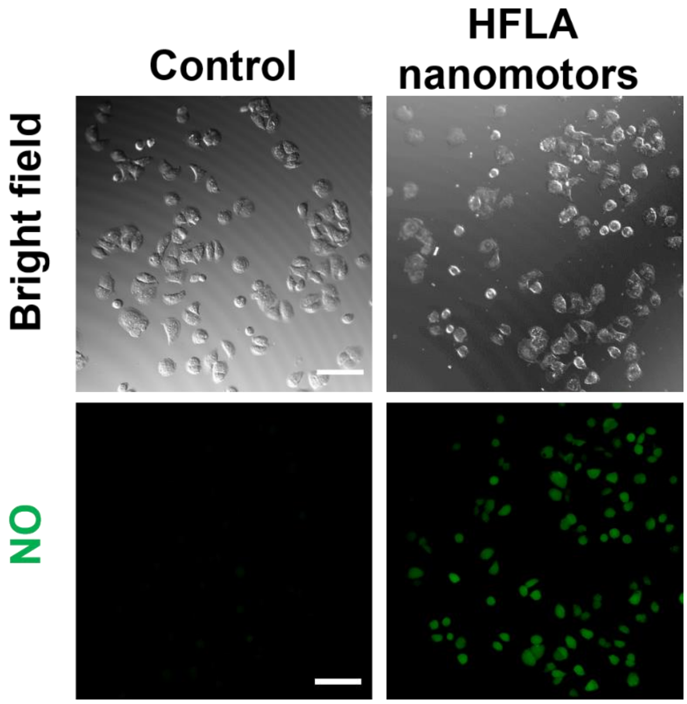

Figure S32. Intracellular NO detection. CLSM images recording the intracellular NO production using the NO fluorescent probe DAF-FM DA for (a) control, and (b) HFLA nanomotors, (Scale bar=100 μm).

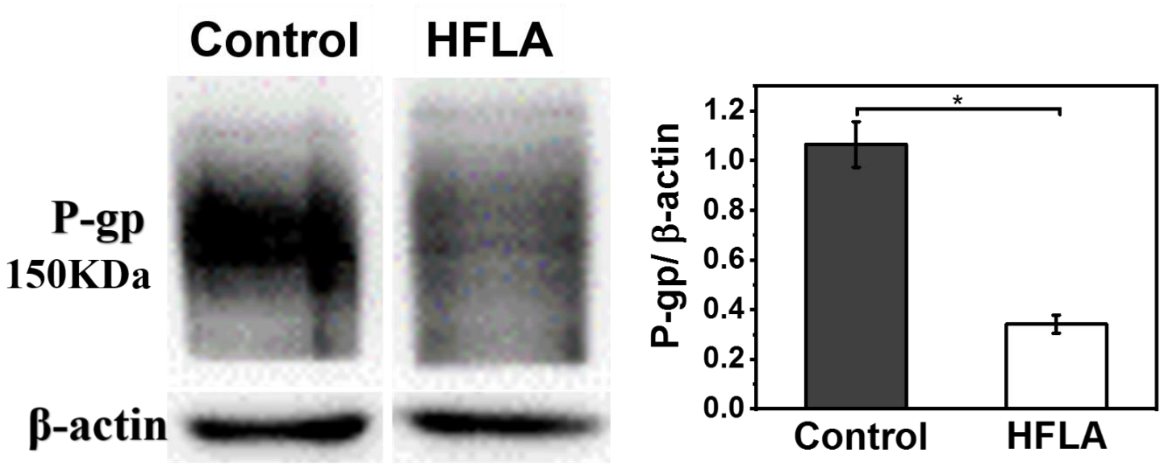

Figure S33. The western blot test. Expression levels of P-gp in MCF-7/ADR cells (a) without treatment and (b) HFLA-treated and their corresponding quantitative statistical analysis. Asterisk (*) denotes statistical significance between bars (*$p < 0.05$) using one-way ANOVA analysis. Experimental data are mean±s.d. of samples in a representative experiment ($n = 3$).

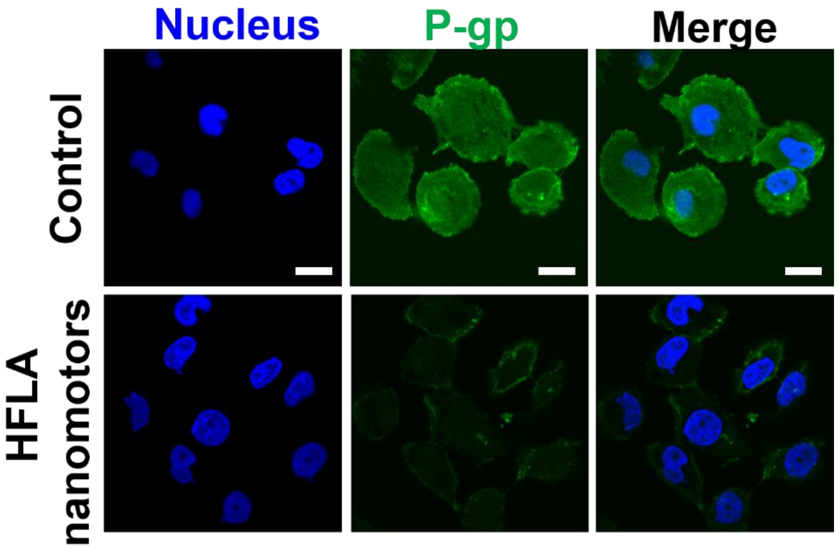

Figure S34. P-gp expression test. P-gp expression of MCF-7/ADR cells using immunofluorescence staining for control, and HFLA nanomotors (Scale bar=20 μm).

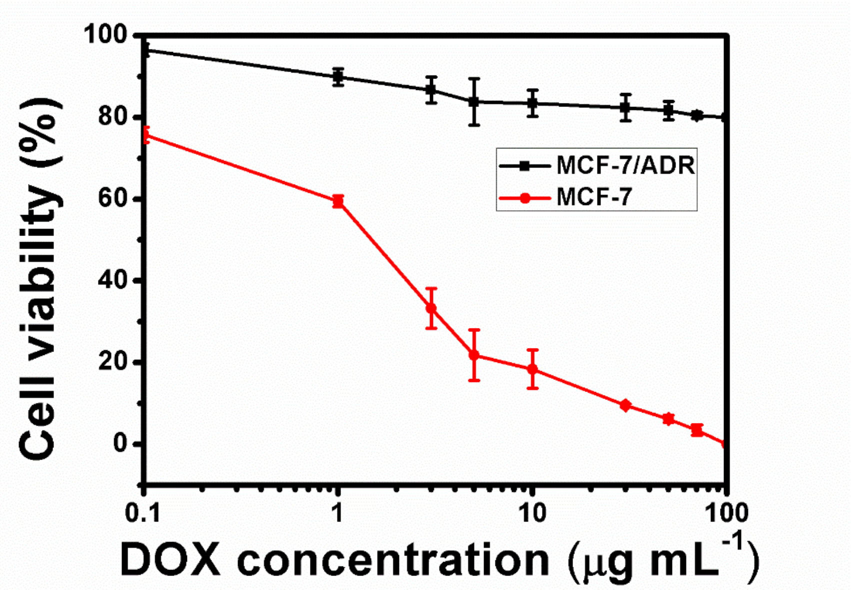

Figure S35. Cell viabilities of MCF-7 and MCF-7/ADR under different concentrations of DOX. Experimental data are mean±s.d. of samples in a representative experiment (n = 5).

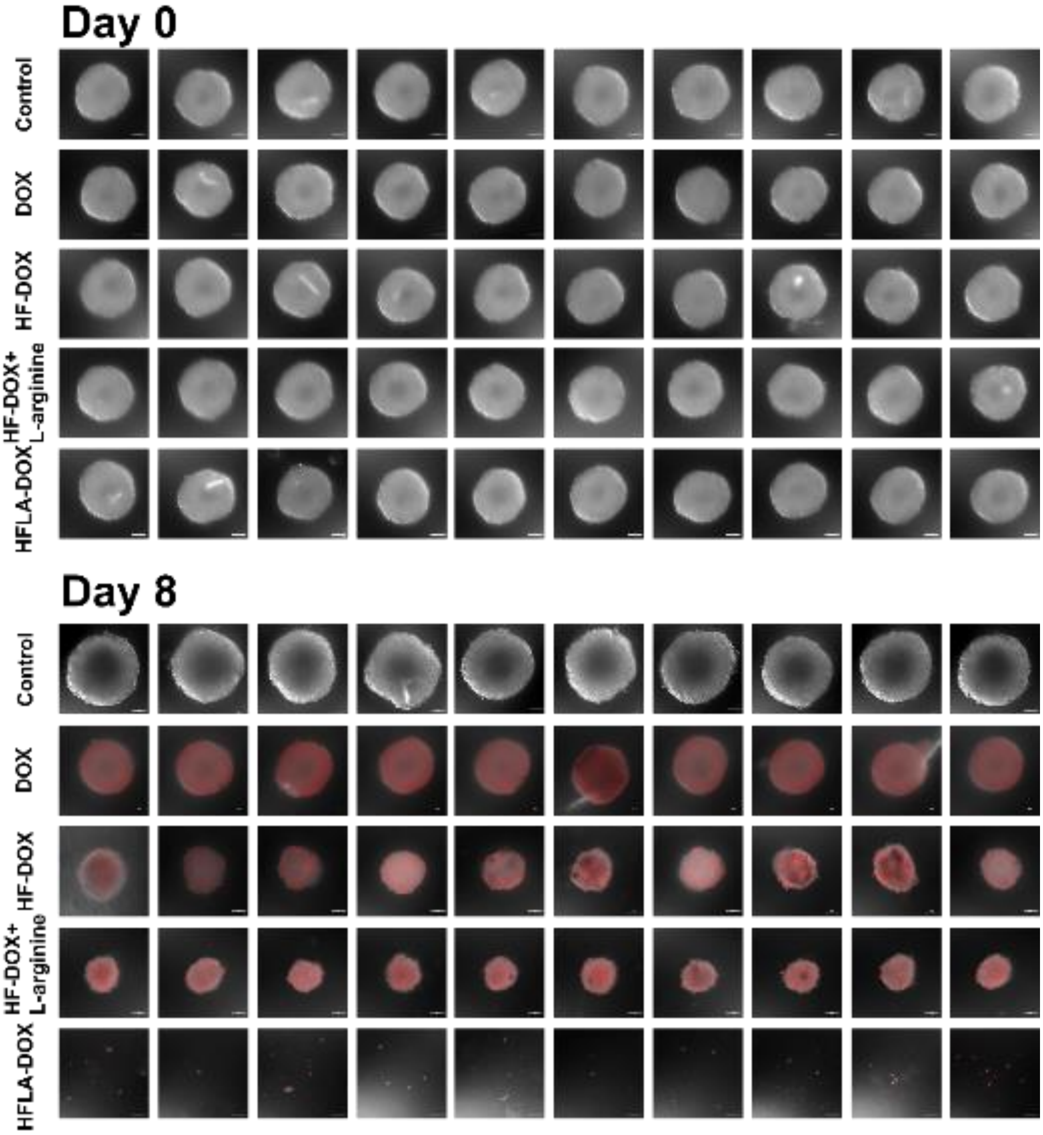

Figure S36. Elimination of 3D MTSs. The image of elimination of 3D MTSs treated under different conditions: control, DOX, HF-DOX, HF-DOX+L-arginine, and HFLA-DOX nanomotors for 0 and 8 days, (Scale bar: 100 μm).

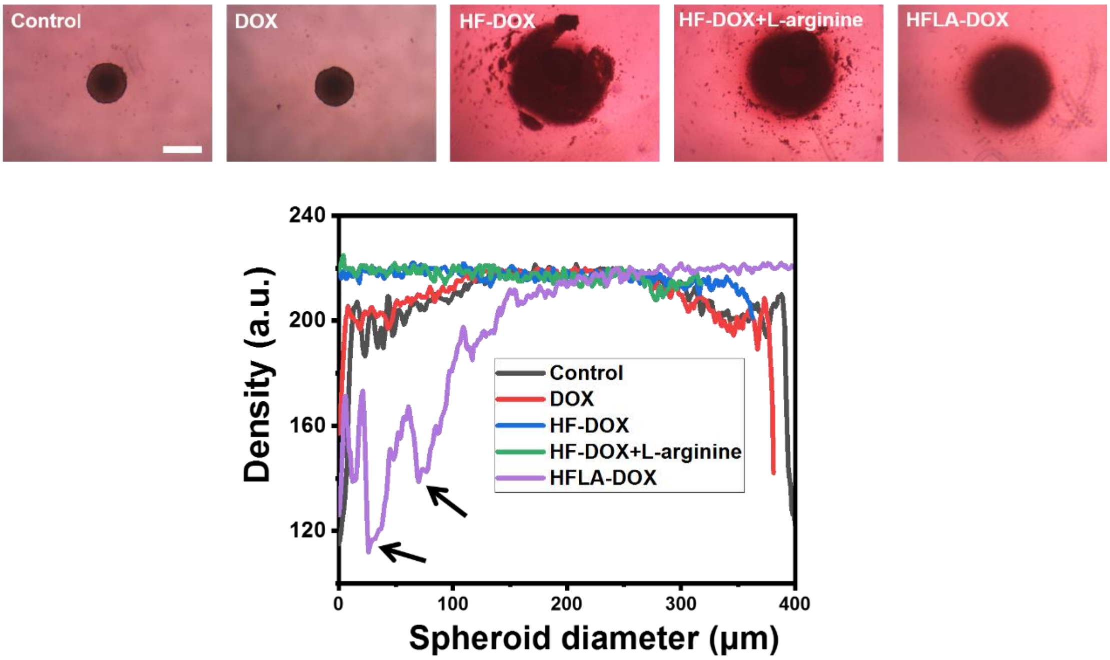

Figure S37. Representative pictures and cellular density within spheroids treated as indicated for 8 days (Scale bar: 50 μm).

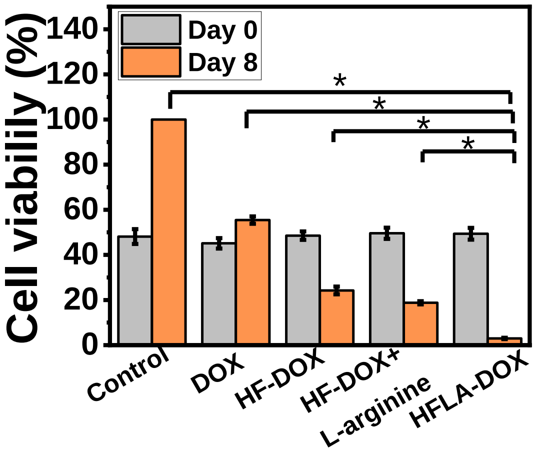

Figure S38. The cell viability of DOX, HF-DOX, HF-DOX+L-arginine, and HFLA-DOX in day 0 and day 8, respectively. Asterisk (*) denotes statistical significance between bars (*$p < 0.05$) using one-way ANOVA analysis. Experimental data are mean±s.d. of samples in a representative experiment (n = 10).

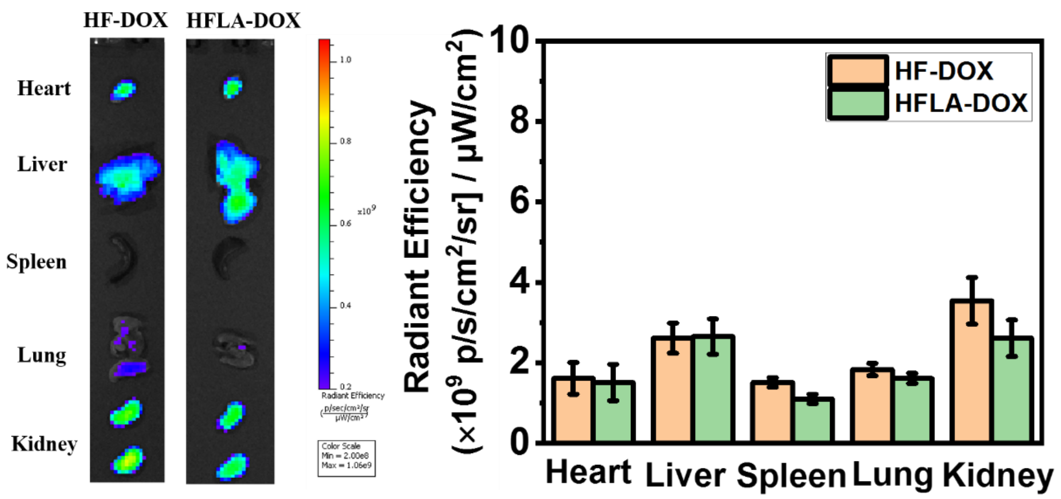

Figure S39. Biodistribution of Cy5.5 labeled HF-DOX and HFLA-DOX in organs of nude mice at 24 h after injection. Experimental data are mean±s.d. of samples in a representative experiment (n = 3).

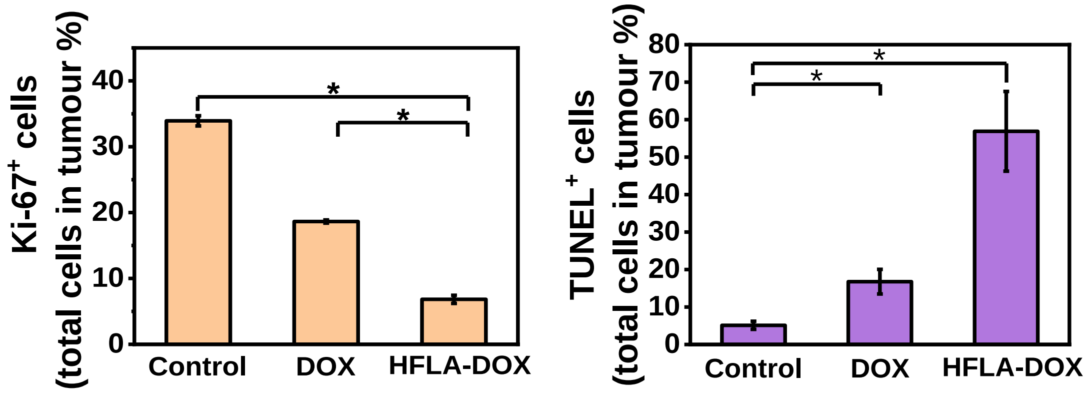

Figure S40. Statistical assay for Ki-67 and TUNEL results.

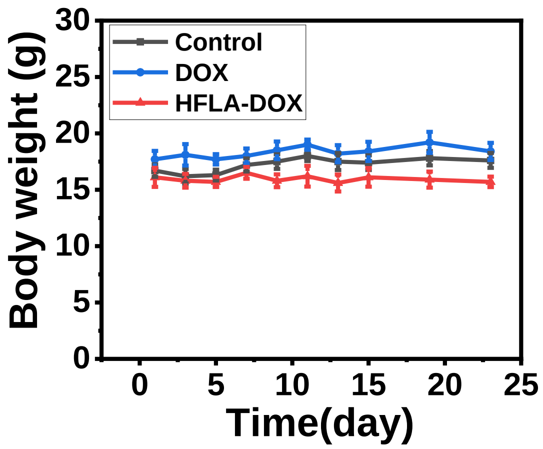

Figure S41. The body weight of the mice during treatment process. Experimental data are mean±s.d. of samples in a representative experiment (n = 5).

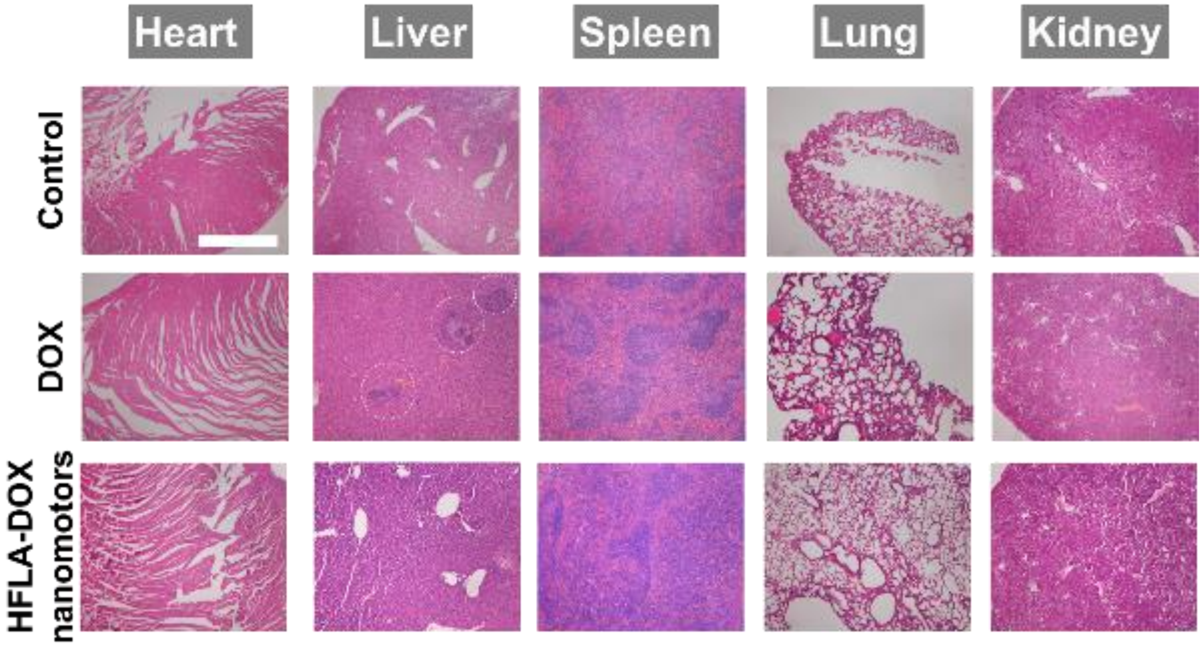

Figure S42. Toxicity evaluations of nanomotors. The tissues of organs (heart, liver, spleen, lung and kidney) of the mice during different treatment process (control, DOX and HFLA-DOX nanomotors) were first stained with hematoxylin and eosin (H&E); (Scale bar: 100 μm).

Table S1. Summary of the function of NO in chemotherapy.

NO source	Stimulus source	Penetration						g#	h#	Ref
		a*	b*	c*	d*	e*	f*			
NO-wLED	LED irradiation,	×	×	×	×	×	×	×	√	Adv. Mater. 2020 [19]
pPTX/pCD-pSNO	Redox-responsive	×	×	×	×	×	×	×	√	Adv. Funct. Mater. 2020 [20]
GCZ@M	pH/ultrasound	×	×	×	×	×	×	×	√	Biomaterials 2020 [21]
PEG-PPS-GSNO@DOX	Glutathione (GSH)	×	×	×	×	×	×	√	√	Acta Biomater. 2020 [22]
NOPD-DOXO	Visible light	×	×	×	×	×	×	√	√	Biomater. Sci. 2020 [23]
NanoNO	pH	√	×	×	×	×	×	×	√	Nat. Nanotechnol. 2019 [24]
DN@MSN	pH	√	×	×	×	×	√	×	√	Nano Lett. 2019 [25]
$_{AD}$Au@CuS YSNPs	NIR	×	×	×	×	×	×	√	√	Nano Lett. 2019 [26]
BNN6@FAPPs	NIR	×	×	×	×	×	×	√	√	Acta Biomater. 2019 [27]
NO-M@DOX	GSH	×	×	×	×	×	×	√	√	ACS Macro Lett. 2019 [28]
SDC@NS	GSH or NIR	×	×	×	×	×	×	×	√	J. Mater. Chem. B 2019 [29]
S-nitrosothiol MIPs	GSH	×	×	×	×	×	×	×	√	Colloid. Surface. B 2019 [30]
CuS@MSN-NO	NIR	×	×	×	×	×	×	√	√	Chem. Phys. Chem. 2019 [31]
DETA/NO	H_2O	×	×	×	×	×	×	×	√	Molecules 2019 [32]
PEG-b-NO-Dex	GSH	√	×	×	×	×	×	×	√	Nano Lett. 2018 [33]
GSNO-HANPs	Ascorbic acid	×	×	×	×	×	×	×	√	ACS Appl. Mater. Inter. 2018 [34]
p(GD-Az-JSK)/DOX	GSH	×	×	×	×	×	×	×	√	Nanoscale 2018 [35]
Pt(IV)-NO/Emicelle	UV	×	×	×	×	×	×	×	√	Bioconjugate Chem. 2018 [36]
F-nanogel (DOX)	pH	×	×	×	×	×	×	×	√	Eur. Polym. J. 2018 [37]
$_{L}$-Arg-HMON-GOx	H_2O_2	×	×	×	×	×	×	×	√	Angew. Chem. Int. Ed. 2017 [38]
PTNG	NIR	×	×	×	×	×	×	√	√	Adv. Funct. Mater. 2017 [39]
TPGS-NO_3	The reductive	√	×	×	×	×	×	√	√	J. Control. Release 2017

	tumor environment									[40]
UCNPs(DOX) @CS-RBS	NIR	×	×	×	×	×	×	×	√	Acta Biomater. 2017 [41]
RBS-T-UCNPs	NIR	×	×	×	×	×	×	×	√	Sci. Bull. 2017 [42]
DOX−NOP1	Visible light	×	×	×	×	×	×	√	√	ACS Med. Chem. Lett. 2017 [43]
Peptide-HMSN-LA	US	√	×	×	×	×	×	×	√	ACS Nano 2016 [44]
mPEG-PLGA-BNN6	UV	×	×	×	×	×	×	√	√	ACS Appl. Mater. Inter. 2016 [45]
mPEG-PEI-PLLA-PTX-NO	H_2O	×	×	×	×	×	×	×	√	RSC. Adv. 2016 [46]
DNIC2	2-Mercaptoethanol	×	×	×	×	×	×	×	√	Inorg. Chem. 2016 [47]
GSNO-MNPs	pH	×	×	×	×	×	×	×	√	Colloid. Surface. B 2016 [48]
Acridone carboxamide derivatives	H_2O	×	×	×	×	×	×	√	√	Bioorg. Chem. 2016 [49]
HMs-CPT-11-DETA NONOate	pH	×	×	×	×	×	×	√	√	Angew. Chem. Int. Ed. 2015 [50]
PEG-USMSs-SNO	X-ray	×	×	×	×	×	×	×	√	Angew. Chem. Int. Ed. 2015 [51]
RBS-UCNPs	NIR	×	×	×	×	×	×	√	√	Adv. Funct. Mater. 2015 [52]
SNO-DOX	pH	×	×	×	×	×	×	×	√	Chem. Commun., 2015 [53]
NO-Si-DETA-cisplatin-AMS	NO gas	×	×	×	×	×	×	×	√	J. Inorg. Biochem. 2015 [54]
DOX&TNO$_3$	GSH	×	×	×	×	×	×	×	√	Mol. Pharm. 2014 [55]
Multi-arm polymer-JSK analogue 1 conjugate	H_2O	×	×	×	×	×	×	×	√	Biomaterials 2012 [56]
Furoxan/DDB	H_2O	×	×	×	×	×	×	√	√	Bioorg. Med. Chem. Lett. 2012 [57]
NO-DOXOs	H_2O	×	×	×	×	×	×	√	√	ACS Med. Chem. Lett. 2011 [58]
DETANONOate	pH	×	×	×	×	×	×	√	√	Nitric Oxide 2009 [59]
NCX-4040	Thiols	×	×	×	×	×	×	√	√	J. Transl. Med. 2008

										[60]
NCX-4016	Esterases	×	×	×	×	×	×	√	√	Proc. Natl. Acad. Sci. USA 2006 [61]
HFLA-DOX	ROS	√	√	√	√	√	√	√	√	This work

a* Vascular permeability
b* Promote endocytosis
c* Intracellular movement
d* Escape from lysosome
e* Golgi efflux
f* Induce tumor collagen depletion
g# Reversal of multidrug resistance
h# Anticancer activity

Table S2. The comparison of the NO release, motion and $ONOO^-$ production among different samples.

	NO release	Motion ability	$ONOO^-$
HF	×	×	×
HF+L-arginine	√	×	√
HFLA	√	√	√
HFLA+uric acid	√	√	×

Table S3. Half maximal inhibitory concentration test. Half maximal inhibitory concentration (IC50) of MCF-7 and MCF-7/ADR cells treated with free DOX.

**Cell lines**	**IC50/μM**
**MCF-7**	2.6
**MCF-7/ADR**	>100

Other Supplementary Material for this manuscript includes the following:
Movie S1 (.avi format). Motion behavior of nanomotors under MCF-7/ADR cellular condition.
Movie S2 (.avi format). Motion behavior of nanomotors under 3D MTSs condition.
Movie S3 (.avi format). Motion behavior of HFLA nanomotors under MCF-7/ADR cellular environment treated with total NOS inhibitors.
Movie S4 (.avi format). 3D movie for cellular uptake testing targeting ability.
Movie S5 (.avi format). 3D movie for cellular uptake.
Movie S6 (.avi format). 3D movie for MCF-7/ADR cellular uptake after being incubated with different endocytic inhibitors.
Movie S7 (.avi format). Motion behavior of HFLA-DOX nanomotors under MCF-7/ADR cellular environment (captured by optical microscope).
Movie S8 (.avi format). Motion behavior of HFLA-DOX nanomotors under RAW246.7 cellular environments.
Movie S9 (.avi format). Motion behavior of HFLA-DOX nanomotors+uric acid in MCF-7/ADR cellular environment.

References

[1] X. Wang, J. Li, Y. X. Wang, L. Koenig, A. Gjyrezi, P. Giannakakou, E.H. Shin, M. Tighiouart, Z. G. Chen, S. M. Nie, D. M. Shin, *ACS Nano* **2011**, *5*, 6184.
[2] T. H. Tran, B. C. Bae, Y. K. Lee, K. Na, K. M. Huha, *Carbohyd. Polym.* **2013,** *92*, 1615.
[3] X. Wang, J. Li, Y. Q. Wang, K. J. Cho, G. Kim, A. Gjyrezi, L. Koenig, P. Giannakakou, H. J. C. Shin, M. Tighiouart, S. M. Nie, Z. G. Chen, D. M. Shin, *ACS Nano* **2009**, *3*, 3165.
[4] A. H. Ennor, J. F. Morrison, H. Rosenberg, *BBA-Bioenergetics* **1955**, *18*, 281.
[5] M. M. Wan, H. Chen, Q. Wang, Q. Niu, P. Xu, Y. Q. Yu, T. Y. Zhu, C. Mao, J. Shen, *Nat. Commun.* **2019**, *10*, 966.
[6] L. L. Teng, G. S. Song, Y. C. Liu, X. Y. Han, Z. Li, Y. J. Wang, S. Y. Huan, X. B. Zhang, W. H. Tan, *J. Am. Chem. Soc.* **2019**, *141*, 13572.
[7] J. Q. Wang, D. S. Ma, Q. Lu, S. X. Wu, G. Y. Lee, L. A. Lane, B. Li, L. Quan, Y. Q. Wang, S. M. Nie, *Nanoscale* **2015**, *7*, 15185.
[8] J. C. Dupin, D. Gonbeau, P. Vinatier, A. Levasseur, *Phys. Chem. Chem. Phys*. **2000**, *2*, 1319.
[9] G. Ruan, S. S. Feng, *Biomaterials* **2003**, *24*, 5037.
[10] K. H. Leung, K. Chakraborty, A. Saminathan, Y. Krishnan, *Nat. Nanotechnol.* **2019**, *14*, 76.
[11] H. J. Jang, S. R. Ryoo, K. Kostarelos, S. W. Han, D. H. Min, *Biomaterials* **2013**, *34*, 3503.
[12] Q. Zhou, S. Q. Shao, J. Q. Wang, C. H. Xu, J. J. Xiang, Y. Piao, Z. X. Zhou, Q. S. Yu, J. B. Tang, X. R. Liu, Z. H. Gan, R. Mo, Z. Gu, Y. Q. Shen, *Nat. Nanotechnol.* **2019**, *14*, 799.
[13] T. P. Szatrowski, C. F. Nathan, *Cancer Res.* **1991**, *51*, 794.
[14] Y. Feng, S. Yu, T. K. R. Lasell, A. P. Jadhav, E. Macia, P. Chardin, P. Melancon, M. Roth, T. Mitchison, T. Kirchhausen, *Proc. Natl. Acad. Sci. USA* **2003**, *100*, 6469.
[15] J. J. Ma, P. J. Li, W. W. Wang, S. H. Wang, X. T. Pan, F. R. Zhang, S. S. Li, S. Liu, H. Y. Wang, G. Gao, B. L. Xu, Q. P. Yuan, H. Y. Shen, H. Y. Liu, *ACS Nano* **2018**, *12*, 9022.
[16] S. Li, Z. G. Su, M. J. Sun, Y. Y. Xiao, F. Cao, A. W. Huang, H. Y. Li, Q. E. Ping, C. Zhang, *Int. J. Pharm.* **2012**, *436*, 248.
[17] Q. Zhou, S. Q. Shao, J. Q. Wang, C. H. Xu, J. J. Xiang, Y. Piao, Z. X. Zhou, Q. S. Yu, J. B. Tang, X. R. Liu, Z. H. Gan, R. Mo, Z. Gu, Y. Q. Shen, *Nat. Nanotechnol.* **2019**, *14*, 799.
[18] N. Bertrand, J. Wu, X. Y. Xu, N. Kamaly, O. C. Farokhzad, *Adv. Drug Deliver. Rev.* **2014**, *66*, 2.
[19] B. Li, P. Ji, S. Y. Peng, P. Pan, D. W. Zheng, C. X. Li, Y. X. Sun, X. Z. Zhang, *Adv. Mater.* **2020**, 2000376.
[20] J. Kim, L. F. Sestito, S. Im, W. J. Kim, S. N. Thomas, *Adv. Funct. Mater.* **2020**, 1908788.
[21] J. An, Y. G. Hu, C. Li, X. L. Hou, K. Cheng, B. Zhang, R. Y. Zhang, D. Y. Li, S. J. Liu, B. Liu, D. Zhu, Y. D. Zhao, *Biomaterials* **2020**, *230*, 119636.
[22] W. Wu, M. Chen, T. Luo, Y. Fan, J. Q. Zhang, Y. Zhang, Q. Y. Zhang, A. S. Minet, C. Gaucher, X. F. Xia, *Acta Biomater.* **2020**, *103*, 259.
[23] F. Sodano, R. J. Cavanagh, A. K. Pearce, L. Lazzarato, B. Rolando, A. Fraix, T. F. Abelha, C. E. Vasey, C. Alexander, V. Taresco, S. Sortino, *Biomater. Sci.* **2020**, *8*, 1329.
[24] Y. C. Sung, P. R. Jin, L. A. Chu, F. F. Hsu, M. R. Wang, C. C. Chang, S. J. Chiou, J. T. Qiu, D. Y. Gao, C. C. Lin, Y. S. Chen, Y. C. Hsu, J. Wang, F. N. Wang, P. L. Yu, A. S. Chiang, A. Y. T. Wu, J. J. S. Ko, C. P. K. Lai, T. T. Lu, Y. Chen, *Nat. Nanotechnol.* **2019**, *14*, 1160.
[25] X. Dong, H. J. Liu, H. Y. Feng, S. C. Yang, X. L. Liu, X. Lai, Q. Lu, J. F. Lovell, H. Z. Chen, C. Fang, *Nano Lett.* **2019**, *19*, 997.
[26] L. Wang, Y. Chang, Y. L. Feng, X. Li, Y. Cheng, H. Jian, X. M. Ma, R. X. Zheng, X. Q. Wu, K. Q. Xu, H. Y. Zhang, *Nano Lett.* **2019**, *19*, 6800.
[27] G. Q. Wei, G. Yang, B. C. Wei, Y. Wang, S. B. Zhou, *Acta Biomater.* **2019**, *100*, 365.

[28] L. L. Gao, B. Dong, J. M. Zhang, Y. Chen, H. S. Qiao, Z. H. Liu, E. P. Chen, Y. Q. Dong, C. J. Cao, D. C. Huang, W. Chen, *ACS Macro Lett.* **2019**, *8*, 1552.
[29] C. Zhang, Q. Q. Li, Y. Zhao, H. Liu, S. L. Song, Y. Q. Zhao, Q. Lin, Y. L. Chang, *J. Mater. Chem. B* **2019**, *7*, 548.
[30] T. W. Liu, Z. Y. Qiao, J. L. Wang, P. Zhang, Z. D. Zhang, D. S. Guo, X. L. Yang, *Colloid. Surface. B* **2019**, *173*, 356.
[31] X. Y. Jiao, Z. M. Wang, F. Wang, Y. Q. Wen, *Chem. Phys. Chem.* **2019**, *20*, 3271.
[32] S. Waheed, R. Y. Cheng, Y. Casablanca, G. L. Maxwell, D. A. Wink, V. Syed, *Molecules* **2019**, *24*, 3722.
[33] V. G. Deepagan, H. Ko, S. Kwon, N. V. Rao, S. K. Kim, W. Um, S. Lee, J. Min, J. Lee, K. Y. Choi, S. Shin, M. Suh, J. H. Park, *Nano Lett.* **2018**, *18*, 2637.
[34] D. E. Kim, C. W. Kim, H. J. Lee, K. H. Min, K. H. Kwack, H. W. Lee, J. Bang, K. Chang, S. C. Lee, *ACS Appl. Mater. Inter.* **2018**, *10*, 26870.
[35] J. M. Zhang, H. J. Song, S. L. Ji, X. M. Wang, P. S. Huang, C. G. Zhang, W. W. Wang, D. L. Kong, *Nanoscale* **2018**, *10*, 4179.
[36] S. Pramanick, J. Kim, J. Kim, G. Saravanakumar, D. Park, W. J. Kim, *Bioconjugate Chem.* **2018**, *29*, 885.
[37] E. B. Kang, G. B. Lee, I. In, S. Y. Park, *Eur. Polym. J.* **2018**, *101*, 96.
[38] W. P. Fan, N. Lu, P. Huang, Y. Liu, Z. Yang, S. Wang, G. C. Yu, Y. J. Liu, J. K. Hu, Q. J. He, J. L. Qu, T. F. Wang, X. Y. Chen, *Angew. Chem. Int. Ed.* **2017**, *56*, 1229.
[39] R. R. Guo, Y. Tian, Y. J. Wang, W. L. Yang, *Adv. Funct. Mater.* **2017**, *27*, 1606398.
[40] M. X. Yin, S. W. Tan, Y. L. Bao, Z. P. Zhang, *J. Control. Release* **2017**, *258*, 108.
[41] L. J. Tan, R. Huang, X. Q. Li, S. P. Liu, Y. M. Shen, *Acta Biomater.* **2017**, *57*, 498.
[42] X. Zhang, Z. Guo, J. Liu, G. Tian, K. Chen, S. C. Yu, Z. J. Gu, *Sci. Bull.* **2017**, *62*, 985.
[43] K. Chegaev, A. Fraix, E. Gazzano, G. E. F. Abd-Ellatef, M. Blangetti, B. Rolando, S. Conoci, C. Riganti, R. Fruttero, A. Gasco, S. Sortino, *ACS Med. Chem. Lett.* **2017**, *8*, 361.
[44] K. Zhang, H. X. Xu, X. Q. Jia, Y. Chen, M. Ma, L. P. Sun, H. R. Chen, *ACS Nano* **2016**, *10*, 10816.
[45] J. Fan, Q. J. He, Y. Liu, F. W. Zhang, X. Y. Yang, Z. Wang, N. Lu, W. P. Fan, L. S. Lin, G. Niu, N. Y. He, J. B. Song, X. Y. Chen, *ACS Appl. Mater. Inter.* **2016**, *8*, 13804.
[46] J. Fan, J. B. Song, Y. J. Liu, G. C. Yu, Y. Ma, Y. Deng, N. Y. He, F. W. Zhang, *RSC Adv.* **2016**, *6*, 105871.
[47] S. C. Wu, C. Y. Lu, Y. L. Chen, F. C. Lo, T. Y. Wang, Y. J. Chen, S. S. Yuan, W. F. Liaw, Y. M. Wang, *Inorg. Chem.* **2016**, *55*, 9383.
[48] H. J. Lee, D. E. Kim, D. J. Park, G. H. Choi, D. N. Yang, J. S. Heo, S. C. Lee, *Colloid. Surface. B* **2016**, *146*, 1.
[49] V. V. S. R. Prasad, G. D. Reddy, I. Kathmann, M. Amareswararao, G. J. Peters, *Bioorg. Chem.* **2016**, *64*, 51.
[50] M. F. Chung, H. Y. Liu, K. J. Lin, W. T. Chia, H. W. Sung, *Angew. Chem. Int. Ed.* **2015**, *54*, 9890.
[51] W. P. Fan, W. B. Bu, Z. Zhang, B. Shen, H. Zhang, Q. J. He, D. L. Ni, Z. W. Cui, K. L. Zhao, J. W. Bu, J. L. Du, J. N. Liu, J. L. Shi, *Angew. Chem. Int. Ed.* **2015**, *54*, 14026.
[52] X. Zhang, G. Tian, W. Y. Yin, L. M. Wang, X. P. Zheng, L. Yan, J. X. Li, H. R. Su, C. Y. Chen, Z. J. Gu, Y. L. Zhao, *Adv. Funct. Mater.* **2015**, *25*, 3049.
[53] M. R. Wang, S. J. Chiu, H. C. Choua, T. M. Hu, *Chem. Commun.* **2015**, *51*, 15649.
[54] I. Munaweera, Y. Shi, B. Koneru, A. Patel, M. H. Dang, A. J. D. Pasqua, K. J. Balkus Jr, *J. Inorg. Biochem.* **2015**, *153*, 23.
[55] Q. L. Song, S. W. Tan, X. T. Zhuang, Y. Y. Guo, Y. D. Zhao, T. T. Wu, Q. Ye, L. Q. Si, Z. P. Zhang, *Mol. Pharm.* **2014**, *11*, 4118.
[56] S. F. Duan, S. Cai, Q. H. Yang, M. L. Forrest, *Biomaterials* **2012**, *33*, 3243.

[57] X. B. Tang, X. K. Gu, H. Ai, G. J. Wang, H. Peng, Y. S. Lai, Y. H. Zhang, *Bioorg. Med. Chem. Lett.* **2012**, *22*, 801.
[58] K. Chegaev, C. Riganti, L. Lazzarato, B. Rolando, S. Guglielmo, I. Campia, R. Fruttero, A. Bosia, A. Gasco, *ACS Med. Chem. Lett.* **2011**, *2*, 494.
[59] S. Huerta-Yepez, M. Vega, S.E. Escoto-Chavez, B. Murdock, T. Sakai, S. Baritaki, B. Bonavida, *Nitric Oxide* **2009**, *20*, 39.
[60] A. Bratasz, K. Selvendiran, T. Wasowicz, A. Bobko, V. V. Khramtsov, L. J. Ignarro, P. Kuppusamy, *J. Transl. Med.* **2008,** *6*, 9.
[61] A. Bratasz, N. M. Weir, N. L. Parinandi, J. L. Zweier, R. Sridhar, L. J. Ignarro, P. Kuppusamy, *Proc. Natl. Acad. Sci. USA* **2006**, *103*, 3914.
